# *Bacillus subtilis* forms twisted cells with cell wall integrity defects upon removal of the molecular chaperones DnaK and trigger factor

**DOI:** 10.3389/fmicb.2022.988768

**Published:** 2023-01-16

**Authors:** Judith Matavacas, Joel Hallgren, Claes von Wachenfeldt

**Affiliations:** Department of Biology, Lund University, Lund, Sweden

**Keywords:** chaperone, protein homeostasis, cell shape, protein aggregation, DnaK, trigger factor, *Bacillus subtilis*

## Abstract

The protein homeostasis network ensures a proper balance between synthesis, folding, and degradation of all cellular proteins. DnaK and trigger factor (TF) are ubiquitous bacterial molecular chaperones that assist in protein folding, as well as preventing protein misfolding and aggregation. In *Escherichia coli*, DnaK and TF possess partially overlapping functions. Their combined depletion results in proteostasis collapse and is synthetically lethal at temperatures above 30°C. To increase our understanding on how proteostasis is maintained in Gram-positive bacteria, we have investigated the physiological effects of deleting *dnaK* and *tig* (encoding for DnaK and TF) in *Bacillus subtilis*. We show that combined deletion of *dnaK* and *tig* in *B. subtilis* is non-lethal, but causes a severe pleiotropic phenotype, including an aberrant twisted and filamentous cell morphology, as well as decreased tolerance to heat and to cell wall active antibiotics and hydrolytic enzymes, indicative of defects in cell wall integrity. In addition, cells lacking DnaK and TF have a much smaller colony size due to defects in motility. Despite these physiological changes, we observed no major compromises in important cellular processes such as cell growth, FtsZ localization and division and only moderate defects in spore formation. Finally, through suppressor analyses, we found that the wild-type cell shape can be partially restored by mutations in genes involved in metabolism or in other diverse cellular processes.

## 1. Introduction

To carry out their specific functions, most proteins need to adopt a well-defined three-dimensional structure: their native state. It is fundamental for any cell to contain enough correctly folded proteins, so that essential biological processes can be performed, and to prevent accumulation of misfolded and aggregated proteins which can interfere with these processes. To maintain a functional proteome, cells have evolved a complex regulatory network that operates primarily to ensure a dynamic equilibrium between protein synthesis, folding, transport, and degradation. This equilibrium is referred to as protein homeostasis or proteostasis (Balch et al., 2008; Powers et al., 2009; Schramm et al., 2020; Matavacas and von Wachenfeldt, 2022).

While some proteins fold spontaneously into their native state after being synthesized, others require assistance by evolutionary conserved molecular chaperones. Chaperones assist in the folding of newly synthesized proteins and prevent formation and accumulation of protein aggregates during and after translation, and are thus key to proteostasis maintenance, especially in response to proteotoxic stress conditions that challenge protein stability (Mogk et al., 2011; Balchin et al., 2016).

In prokaryotes, the Hsp70 homolog DnaK and trigger factor (TF) are two of the major cytosolic chaperones that participate in the protein folding process (Hartl and Hayer-Hartl, 2002). TF in its monomeric form is associated to the ribosome through its N-terminal domain and interacts with nascent polypeptide chains *via* its C-terminal substrate-binding domain during translation, stabilizing them for subsequent folding, while preventing misfolding and aggregation (Stoller et al., 1995; Ferbitz et al., 2004; Singhal et al., 2015). The N- and C-terminal domains of TF are linked by a peptidyl-prolyl isomerase domain. A wide range of protein substrates can be stabilized by the TF, and this is probably due to its structural flexibility and the promiscuous clamp-like structure formed by its C-terminal domain (Martinez-Hackert and Hendrickson, 2009; Saio et al., 2014). TF can also be present in the cytosol in a dimeric form, not bound to the ribosome, also exhibiting protein folding assistance (Saio et al., 2018). DnaK is monomeric and contains an N-terminal ATPase domain and a C-terminal substrate-binding domain (Zhu et al., 1996). The substrate-binding domain enables interaction with non-native protein species that exhibit exposed hydrophobic peptide stretches (Rudiger et al., 1997; Mogk et al., 1999; Calloni et al., 2012). Together with its molecular co-chaperone DnaJ (Hsp40) and the nucleotide exchange factor GrpE, DnaK stabilizes substrates through several binding and release cycles fueled by ATP-hydrolysis, decreasing the protein folding rate and preventing aberrant premature folding and aggregation (Liberek et al., 1991; Szabo et al., 1994).

Chaperones like DnaK and TF are ancient and possess evolutionary conserved structures and functions. Remarkably, no additional core chaperones have appeared during billion years of evolution despite proteomes becoming more complex and containing more unstable proteins (Rebeaud et al., 2021). However, apart from their conserved mechanisms, chaperones can also display host-specific roles, and their involvement in proteostasis often varies among organisms. In the case of bacteria, this is illustrated by the different phenotypic effects of chaperone deletion in *Escherichia coli* and *Bacillus subtilis*, two well-studied model organisms for Gram-negative and Gram-positive bacteria, respectively. For instance, DnaK in *E. coli* controls the heat-shock sigma factor σ^32^ (Gamer et al., 1992; Liberek et al., 1992) and is required for growth at high and low temperatures (Paek and Walker, 1987; Bukau and Walker, 1989). In addition, deletion of *dnaK* in *E. coli* leads to defects in growth and cell division, and regulation of the heat-shock response (Paek and Walker, 1987; Bukau and Walker, 1989, 1990). In contrast, the absence of DnaK in *B. subtilis* does not seem to create any major cellular defect at temperatures between 16°C and 52°C (Schulz et al., 1995).

Importantly, DnaK and TF have been shown to possess overlapping functions in *E. coli* (Deuerling et al., 1999; Teter et al., 1999; Deuerling et al., 2003; Genevaux et al., 2004; Calloni et al., 2012), sharing a substrate pool *in vivo* (Deuerling et al., 2003), which might explain why the fraction of newly synthesized polypeptides that interact with DnaK increases in the absence of TF (Deuerling et al., 1999; Teter et al., 1999; Calloni et al., 2012). Furthermore, the contributions of both chaperones to proteostasis maintenance seems to be crucial even at the normal temperature range, since in contrast to the respective single mutants, hundreds of cytosolic proteins appear to aggregate in the *dnaK tig* double mutant at 30°C (Deuerling et al., 1999; Vorderwulbecke et al., 2004), and such double deletion is lethal above 30°C (Deuerling et al., 1999; Teter et al., 1999; Genevaux et al., 2004). In *B. subtilis*, a very brief study of a *dnaK dnaJ tig* triple deletion mutant reported that this strain is highly temperature sensitive and does not grow at ≥53°C, but, unlike the *E. coli dnaK tig* double mutant, cells are viable below this temperature (Reyes and Yoshikawa, 2002), suggesting that the *de novo* folding function is not exclusive of DnaK and TF, but is shared with other systems.

Here, we further examined the phenotypic consequences of deleting *dnaK* and *tig* in *B. subtilis*. We observed that lack of both chaperones causes important pleiotropic phenotypic effects, such as an increased temperature sensitivity, and increased sensitivity to lysozyme and cell wall-active antibiotics. Interestingly, the *dnaK tig* double mutant displayed a twisted and filamentous morphology, in addition to having a remarkably reduced colony size. Our experiments suggest that cell wall integrity is affected by the *dnaK tig* double deletion, which could explain the observed aberrant morphology. We found through suppressor analyses mutations that could partially suppress some of the described phenotypic traits, revealing genes that might be related to or involved in coping with heat, defining cell shape, or ensuring proteostasis.

## 2. Materials and methods

### 2.1. Bacterial strains, plasmids and growth conditions

The *B. subtilis* strains and plasmids used in this work are listed in Tables 1, 2, respectively. *E. coli* TOP10 or DH5α were grown in lysogeny broth (LB) or on LB agar (LA) plates, and used for plasmid amplification and isolation. *B. subtilis* strains were grown on tryptose blood agar base (TBAB), in nutrient sporulation medium with phosphate (NSMP) supplemented with 0.5% glucose (NSMPG) (Winstedt et al., 1998), minimal MG medium (Hoch, 1991) or in LB. The temperature for the growth of all bacterial strains for most experiments was 30°C, unless otherwise stated (with shaking at 200 rpm in baffled flasks, for liquid cultures). The growth medium was supplemented with the following antibiotics when appropriate: ampicillin (100 μg mL^−1^), chloramphenicol (5 μg mL^−1^), kanamycin (5 μg mL^−1^), lincomycin (12.5 μg mL^−1^), and erythromycin (0.5 μg mL^−1^).

**Table 1:** *Bacillus subtilis* strains used in this study.

Strain	Relevant characteristics^a^	Reference^b^
1A1	*trpC2*	BGSC
BKK25470	*trpC2* Δ*dnaK*::*kan*, Km^R^	BGSC
BKE28230	*trpC2* Δ*tig*::*erm*, Em^R^	BGSC
LUW876	Δ*dnaK*::*kan*, Km^R^	This work
LUW901	Δ*tig*::*erm*, Em^R^	This work
LUW878	Δ*dnaK*::*kan* Δ*tig*::*erm*, Km^R^ Em^R^	This work
LUW1173	Δ*tig*::*erm amyE*::P*_tig_*_*tig*, Km^R^ Em^R^ Cm^R^	This work
LUW1175	Δ*dnaK*::*kan* Δ*tig*::*erm amyE*::P*_tig_*_*tig*, Em^R^ Cm^R^	This work
LUW1148	Δ*tig*::*erm amyE*::P*_hyperspank_*_*tig*, Em^R^ Cm^R^	This work
LUW1150	Δ*dnaK*::*kan* Δ*tig*::*erm amyE*::P*_hyperspank_*_*tig*, Km^R^ Em^R^ Cm^R^	This work
LUW896	Δ*dnaK*::*kan amyE*::P*_hyperspank_*_*dnaK*, Km^R^ Cm^R^	This work
LUW907	Δ*dnaK*::*kan* Δ*tig*::*erm amyE*::P*_hyperspank_*_*dnaK*, Km^R^ Em^R^ Cm^R^	This work
LUW1025	*amyE*::P*_hyperspank_*_*zapA*, Cm^R^	This work
LUW1033	Δ*dnaK*::*kan amyE*::P*_hyperspank_*_*zapA*, Km^R^ Cm^R^	This work
LUW1034	Δ*tig*::*erm amyE*::P*_hyperspank_*_*zapA*, Em^R^ Cm^R^	This work
LUW1035	Δ*dnaK*::*kan* Δ*tig*::*erm amyE*::P*_hyperspank_*_*zapA*, Km^R^ Em^R^ Cm^R^	This work
LUW1193	*aprE*::P*_spac_*-*mcherry-mreB,* Cm^R^	This work
LUW1194	Δ*dnaK*::*kan aprE*::P*_spac_*-*mcherry-mreB,* Km^R^ Cm^R^	This work
LUW1195	Δ*tig*::*erm aprE*::P*_spac_*-*mcherry-mreB,* Em^R^ Cm^R^	This work
LUW1196	Δ*dnaK*::*kan* Δ*tig*::*erm aprE*::P*_spac_*-*mcherry-mreB,* Km^R^ Em^R^ Cm^R^	This work
LUW1155	*amyE*::P*_hag_-gfp*, Cm^R^	This work
LUW1179	Δ*dnaK*::*kan amyE*::P*_hag_-gfp*, Km^R^ Cm^R^	This work
LUW1180	Δ*tig*::*erm amyE*::P*_hag_-gfp*, Em^R^ Cm^R^	This work
LUW1188	Δ*dnaK*::*kan* Δ*tig*::*erm amyE*::P*_hag_-gfp*, Km^R^ Em^R^ Cm^R^	This work
LUW1270	*amyE*::P*_const_-gfp*, Cm^R^	This work
LUW1271	Δ*dnaK*::*kan*, *amyE*::P*_const_-gfp*, Km^R^ Cm^R^	This work
LUW1272	Δ*tig*::*erm*, *amyE*::P*_const_-gfp*, Em^R^ Cm^R^	This work
LUW1273	Δ*dnaK*::*kan* Δ*tig*::*erm*, *amyE*::P*_const_-gfp*, Km^R^ Em^R^ Cm^R^	This work
TNVS205	*aprE*::P*_spac_*-*mcherry-mreB,* Cm^R^	Wenzel et al. (2021)
Weiss96	*amyE*::P*_hag_-gfp thrC*::P*_const_-lchAA-mcherry*, Cm^R^ Em^R^	Weiss et al. (2019)
Weiss9	*amyE*::P*_const_-yfp*, Cm^R^	Weiss et al. (2019)

^a^All *B. subtilis* LUW strains are derived from *B. subtilis* 1A1. Therefore, they all contain the *trpC2* auxotrophic marker. Cm^R^, Em^R^, and Km^R^ are the abbreviations for chloramphenicol, erythromycin, and kanamycin resistance, respectively. ^b^BGSC Bacillus Genetic Stock Center, Department of Biochemistry, Ohio State University, Columbus. http://www.bgsc.org/.

**Table 2:** Plasmids used in this study.

Plasmid	Relevant characteristics^a^	Reference
pCW101	Ap^R^, Cm^R^	Engman et al. (2012)
pCW101_sfGFP	Ap^R^, Cm^R^	Engman and von Wachenfeldt (2015)
pCW101_tig	Ap^R^, Cm^R^	This work
pCW101_mNG	Ap^R^, Cm^R^	This work
pCW101_mNG-YirB	Ap^R^, Cm^R^	This work
pCW101_mNG-ZapA	Ap^R^, Cm^R^	This work
pCW101_mNG-dnaK	Ap^R^, Cm^R^	This work
pCW101_dnaK	Ap^R^, Cm^R^	This work

^a^Ap^R^ and Cm^R^ indicates resistance to ampicillin and chloramphenicol, respectively.

### 2.2. Bacterial transformations and DNA manipulations

Molecular biology techniques, including *E. coli* transformations, were performed according to Sambrook and Russell (2001). *B. subtilis* chromosomal DNA extraction, as well as transformation of *B. subtilis* with plasmids or with chromosomal DNA was performed as described by Hoch (1991). The oligonucleotides (primers) used in this work are listed in Supplementary Table S1.

### 2.3. Construction of plasmids

All plasmids were constructed by using *E. coli* strain DH5α or TOP10 and standard molecular cloning techniques. For studies in *B. subtilis* part of the plasmid constructs were integrated into the genome (*amyE* locus) *via* double homologous recombination. Note that all pCW101-derived plasmids below replicate in *E. coli* but not in *B. subtilis*.

Plasmid pCW101_tig was created by PCR amplifying the *tig* gene with its native promoter from *B. subtilis* 1A1 chromosomal DNA with primers TIG1b and TIG4. The resulting 1,634 bp amplicon was digested with PacI and BamHI, and ligated into pCW101 cut with the same restriction enzymes.

Plasmid pCW101_mNG-YirB was created by cloning a gene, synthesized by GenScript, encoding mNeonGreen fused with YirB into pCW101 using HindIII/SalI.

Plasmid pCW101_mNG was created by restriction enzyme digestion of plasmid pCW101_mNG-YirB with HindIII and Bsp1407I. The isolated HindIII/Bsp1407I fragment was cloned into pCW101_sfGFP that had been cut with the same enzymes thereby the *sfgfp* gene was replaced with the gene encoding mNeonGreen.

Plasmid pCW101_mNG-ZapA was created by PCR amplifying the *zapA* gene from *B. subtilis* 1A1 chromosomal DNA with primers ZAPA1 and ZAPA2. The resulting 297 bp amplicon was digested with Bsp1407I and SalI, and ligated into pCW101_mNG cut with the same restriction enzymes. In the mNG-ZapA fusion protein, the C-terminus of mNG is fused *via* a (G)_7_ linker to the N-terminal serine (S2) residue of ZapA.

Plasmid pCW101_mNG-dnaK was created by PCR amplifying the *dnaK* gene from *B. subtilis* 1A1 chromosomal DNA with primers DnaKNeongreen1 and DnaKNeongreen2. The resulting amplicon was digested with Bsp1407I and SphI, and ligated into pCW101_mNG cut with the same restriction enzymes.

To construct pCW101_dnaK, we first PCR amplified the native *dnaK* using DNAK2 and DnaKNeongreen2 primers with 1A1 chromosomal DNA as a template. The *dnaK* amplicon was digested with HindIII and Eco52I, resulting in a 569 bp fragment. In parallel, pCW101_mNG-dnaK was digested with Eco52I and NheI, and the resulting 1,292 bp fragment was isolated. pCW101 was cut with HindIII and NheI, and ligated with the two *dnaK* digested fragments at a 1:3:3 ratio, respectively.

### 2.4. Construction of *Bacillus subtilis* strains

Deletion of the *dnaK* gene was done by transforming the strains with chromosomal DNA from strain BKK25470, selecting for kanamycin resistance. Deletion of the *tig* gene was done by transforming the strains with chromosomal DNA from strain BKE28230, selecting for erythromycin and lincomycin resistance.

To complement the Δ*dnaK* Δ*tig* double mutant strain with the *tig* gene under its native promoter, we first PCR amplified the *tig* fragment (flanked by *amyE* upstream and downstream regions, and also containing the chloramphenicol resistance gene) from plasmid pCW101_tig using primers AMYFRONT1 and AMYBACK3. The resulting 1,610 bp amplicon was used to transform strain LUW901. Transformants were selected for resistance to chloramphenicol. Next, the *dnaK* gene was deleted as explained above, creating strain LUW1175.

To integrate the *tig* gene under the IPTG-inducible P*_hyperspank_* promoter in the *amyE* locus, we PCR amplified three fragments: first, a 2,557 bp fragment from plasmid pCW101 using primers AMYBACK1 and TIG_FRAG1REV (this fragment contained the *amyE* upstream region, the chloramphenicol resistance gene, and the P*_hyperspank_* promoter); second, a 2,101 bp fragment from plasmid pCW101 using primers AMYFRONT1 and TIG_FOR_2 (this fragment contained the *lacI* gene and the *amyE* downstream region); third, a 1,315 bp fragment consisting of the *tig* gene from *B. subtilis* 1A1 chromosomal DNA with primers TIG_FRAG2FOR and TIG_REV_2. Next, a PCR was performed with a mixture containing 0.03 pmol of each fragment, using primers AMYFRONT1 and AMYBACK3, and resulting in a 5,800 bp amplicon. Strain LUW901 was transformed with the 5,800 bp amplicon, and transformants were selected for chloramphenicol resistance. The *dnaK* was then removed as explained above, creating strain LUW1150.

To integrate the *dnaK* gene under the IPTG-inducible P*_hyperspank_* promoter at the *amyE* locus, strain LUW876 was transformed with pCW101_dnaK, selecting transformants for resistance to chloramphenicol. The *tig* gene was then removed as explained above, creating strain LUW907.

To create *B. subtilis* strains expressing the mNeonGreen-ZapA (mNG-ZapA) fusion protein, the desired strains were transformed with the pCW101_mNG-ZapA plasmid that contained mNG-ZapA under the IPTG-inducible P*_hyperspank_* promoter flanked by *amyE* upstream and downstream regions. Transformants were selected for resistance to chloramphenicol. To verify the successful integration of the desired genetic fragments into the *amyE* locus, transformants were grown on plates containing 1% starch (w/v), followed by exposure to iodine:water solution (1:1 v/v). A lack of halo formation confirmed the correct integration.

To create *B. subtilis* strains expressing the mCherry-MreB fusion protein, the desired strains were transformed with chromosomal DNA from strain TNVS205, which contained mCherry-MreB under the IPTG-inducible P*_spac_* promoter in the by *aprE* region. Transformants were selected for resistance to chloramphenicol.

To introduce the P*_hag_-gfp* reporter construct in the *amyE* locus, the appropriate strains were transformed with chromosomal DNA from strain Weiss65. Transformants were selected for resistance to chloramphenicol.

To introduce P_const_-*gfp*, with the constitutive phage SP01 promoter in front of the gene encoding GFPmut2 at the *amyE* locus, we PCR amplified two fragments: the first fragment (containing *amyE*-back, a chloramphenicol resistance gene, and the P_const_ promoter) was amplified using genomic DNA from Weiss9 as template and primers AMYBACK3 and GFPMUT2_1; the second fragment, was amplified (containing the GFPmut2 gene and *amyE*-front) using genomic DNA from Weiss96 as template and primers AMYFRONT1 and GFPMUT2_2. PCR fragments were gel-verified and gel-extracted. Next, a PCR was performed with a mixture containing 0.03 pmol of each fragment, using primers AMYFRONT1 and AMYBACK3, and 5 μl of the resulting amplicon mixture was transformed directly into *B. subtilis* 1A1, resulting in strain LUW1270. Strain LUW1271 and LUW1272 were made by transforming LUW1270 with genomic DNA from LUW876 (Δ*dnaK*::kan) or LUW901 (Δ*tig*::erm), respectively. LUW1273 (Δ*dnaK*::kan Δ*tig*::erm, *amyE*::Pconst-*gfp*) was made by transforming LUW1272 with genomic DNA from LUW876.

All constructs were confirmed by sequencing.

### 2.5. Isolation of aggregated proteins

Twenty-five milliliters bacterial cultures were harvested at an OD_600_ of 1 into centrifuge tubes containing 10 g of crushed ice. Cells were pelleted by centrifugation at 12,000 × *g* for 15 min at 4°C. Pellets were washed in 10 ml cold buffer A (50 mM Tris–HCl, 100 mM NaCl, 10 mM EDTA pH 8.0) at 4°C. Cells were pelleted by centrifugation at 12,000 × *g* for 15 min at 4°C. Pellets were frozen, and dissolved in 0.5 ml buffer A supplemented with Complete EDTA-free Protease Inhibitor (Merck). Samples were sonicated on ice (Vibra Cell disruptor, output control ~20, pulser 3 s) for 5 × 20 s, and then centrifuged at 2,000 × *g* for 10 min at 4°C to remove intact cells. 400 μl of the supernatant were collected and centrifuged at 20,000 × *g* for 45 min at 4°C to pellet membranes and aggregates. Pellets were resuspended in 360 μl of buffer A by brief sonication. Next, 40 μl 10% (v/v) Triton X-100 (in buffer A) was added to the samples, following a 10 min at 4°C. Aggregates were pelleted by centrifugation at 20,000 × *g* for 45 min at 4°C. The resuspension of pellets in buffer A, addition of Triton X-100, and aggregate pelleting was repeated to allow complete removal of contaminating membrane proteins. Pelleted aggregates were washed with 200 μl buffer B [50 mM Tris–HCl, 1 mM EDTA (pH 8.0)] and resuspend in 200 μl buffer B by brief sonication. Samples were analyzed by SDS-PAGE using a 10-well (30 μl) Mini-PROTEAN^®^ TGX™ any kDa precast gel (Bio-Rad), and visualized by staining with Bio-Safe Coomassie G-250 (Bio-Rad). Thermo Scientific™ PageRuler™ Prestained Protein Ladder was used as molecular weight markers.

### 2.6. Phase contrast and fluorescence microscopy

To visualize bacteria from TBAB plates, cells were scraped off from single colonies and gently suspended in 5 μl of phosphate-buffered saline solution (PBS) on a microscope slide coated with a layer of agarose (1% agarose in PBS), protecting it with a cover slip. To visualize bacteria from liquid cultures, strains were grown in 25 ml NSMPG cultures in a shaking water bath. When needed, expression of mNG-ZapA and mCherry-MreB was induced with 50 μM IPTG at OD_600_ of 0.2, or with 100 μM IPTG from the start, respectively. At OD_600_ of 0.6, 1 ml samples were taken from the cultures and centrifuged at 16,000 × *g* for 2 min. After discarding the supernatant, the pellet was resuspended in 200 μl of PBS. Samples were kept on ice and visualized under the microscope as soon as possible. Bacteria were put on microscope slides coated with a layer of agarose (1% agarose in PBS), and protected with a cover slip. The phase contrast and fluorescence micrographs were captured by using a Zeiss Axio Imager.Z2 microscope equipped with X-Cite 120 Illumination (EXFO Photonic Solutions Inc.) and an ORCA-Flash4.0 V2 Digital CMOS camera C11440-22CU (Hamamatsu Photonics). ZEN 2 (blue edition) was used to acquire images, which were saved in TIFF format, and processed with Fiji (Schindelin et al., 2012).

### 2.7. Scanning electron microscopy

Bacterial samples for imaging were taken from the mid-exponential growth phase of liquid cultures in NSMPG. Five hundred microliters of the exponentially growing cells were added to 0.5 ml of 6% glutaraldehyde in growth media. After 15 min of incubation at room temperature, bacteria were pelleted (11,000 rpm for 3 min at room temperature) and washed once with 0.1 M cacodylate buffer (pH 7.4). Then, the samples were pelleted again, resuspended in 0.5 ml of 3% glutaraldehyde in 0.1 M cacodylate buffer (pH 7.4) and kept at 4°C overnight. Next day, a drop of each sample was placed on a (5 × 5 mm) poly-L-lysine treated glass slides and sedimented at 4°C for 5 h. The cells attached to the slides were washed three times with 0.1 M cacodylate buffer (pH 7.4), followed by dehydration through a graded ethanol series [70% (2 × 10 min), 96% (2 × 10 min), and 100% (2 × 15 min)] followed by critical point-drying (LEICA EM CPD300). The dried samples were mounted on SEM specimen stubs and sputter coated with gold (Cesington 108 auto, 45 s, 20 mA). Bacteria were observed using a Hitachi SU3500 scanning electron microscope at 5 kV.

### 2.8. Transmission electron microscopy

Exponentially growing cells were harvested (10,000 × *g*, 5 min), transferred into freshly prepared fixative [2.5% glutaraldehyde in 0.1 M sodium cacodylate buffer (pH 7.4)] and incubated for 12 h at 4°C. Cells were then transferred to 0.1 M sodium cacodylate buffer (pH 7.4). Cells were postfixed in 2% osmium tetroxide in distilled water for 1 h at 4°C. The samples were dehydrated in graded ethanol series and embedded in EPON (Agar 100). Ultrathin sections (50 nm) were cut with a Leica UC7 ultratome with a diamond knife and mounted on pioloform coated copper grids. The sections were stained with uranyl acetate (2%, 30 min) and lead citrate (1%, 4 min), and viewed with a TEM at 100 kV (JEOL JEM 1400 Plus).

### 2.9. Microscopy and tracking of motility in single cells

To visualize motility of single cells in real time, cells were first grown in NSMPG liquid cultures at 30°C until late exponential phase. Then, a drop of 10 × diluted bacterial culture was placed on a microscopy glass coverslip which was encircled with a ring of Parafilm. The coverslip was inverted on a microscopy slide. The ring of Parafilm was used so that the coverslip would not be in contact with the microscopy slide, and that the drop would be hanging. Cells were observed with phase-contrast microscopy, and videos were acquired. Individual cells were manually tracked using the MTrackJ plugin of Fiji (Schindelin et al., 2012). Each track had a duration of 1 to 2 seconds.

### 2.10. Heat tolerance assay and colony size measurements

For the heat tolerance assay, bacteria were inoculated in 15 ml test tubes with 2 ml LB and grown at 30°C, with 200 rpm shaking for about 16 h. Cell yields were measured and then adjusted for all the strains to an OD_600_ of 2.5. Serial dilutions (10^−1^, 10^−3^, 10^−5^, 10^−7^) were performed in phosphate-buffered saline solution (PBS), and 5 μl drops were spotted on TBAB plates. Plates were incubated at 25, 30, 37, 45, 49, or 53°C for 24 h. For the colony size assays, the same culture dilution series was used. 200 μl from the proper dilutions were plated on TBAB plates, and plates were incubated at 37°C for 22 h. Colony diameters were measured using Fiji (Schindelin et al., 2012).

### 2.11. Determination of sporulation frequencies

The bacterial strains to be tested were grown in 25 ml NSMP, at 30°C and with 200 rpm shaking for 48 h. Two samples were taken from each culture, and one of them was heat-treated at 80°C for 10 min. The number of viable cells per milliliter in the untreated samples and the number of spores per milliliter in the heat-treated samples were determined by serially diluting the samples in PBS and plating appropriate dilutions on TBAB plates. Plates were incubated at 30°C overnight. Sporulation frequencies were calculated by dividing the spore count by the viable count.

For investigating sporulation on solid media, strains were streaked for confluent growth on NSMP agar plates, and plates were incubated at 30°C. After 10 days of incubation, scraped off material from plates was observed with phase contrast microscopy. The number of spores and the number of vegetative cells was obtained by using MicrobeJ (Ducret et al., 2016).

### 2.12. Lysozyme sensitivity assay

Cells were grown in triplicates in 25 ml NSMPG cultures, at 30°C and with 200 rpm shaking. When cultures reached an OD_600_ of 0.6, lysozyme solution was added to a final concentration of 15 μg/ml. Growth was monitored by taking samples for OD measurements.

### 2.13. Antibiotic disc diffusion assay

The bacterial strains to be tested were grown in 25 ml NSMPG, at 30°C and with 200 rpm shaking. At OD_600_ of 0.6, 200 μl of cells were spread on TBAB plates. After the plates had dried, antibiotic discs were placed on the plates. Three discs were used per antibiotic and per plate. For vancomycin, 30 μg vancomycin discs (Fluka BioChemika) were used. For chloramphenicol and D-cycloserine, 6 mm paper discs (Whatman) containing 10 μl of antibiotic (10 mg/ml and 16 mg/ml, respectively) were used. Plates with antibiotic discs were incubated at 30°C for 20 h, and the diameter of the growth inhibition zones was measured using Fiji (Schindelin et al., 2012).

### 2.14. Determination of minimal inhibitory concentrations

Bacteria were inoculated in 15 ml test tubes with 2 ml LB and grown at 30°C, with 200 rpm shaking for about 16 h. Cell density were measured and then adjusted for all the strains to an OD_600_ of 2.5. Ten microliters of the adjusted overnight cultures were inoculated in 15 ml test tubes containing 2 ml LB with different antibiotic concentrations (vancomycin or D-cycloserine). Tubes were incubated at 30°C, with 200 rpm shaking for about 16 h. The minimal inhibitory concentration (MIC) was determined as the lowest antibiotic concentration at which no growth was observed.

### 2.15. Whole genome re-sequencing

Chromosomal DNA extraction from strains whose genome was to be re-sequenced was performed by using the QIAamp^®^ DNA Mini Kit (QIAGEN). A 5 mM Tris/HCl (pH 8.5) buffer was used for the elution step. Whole genome re-sequencing was done as described previously (von Wachenfeldt et al., 2021).

## 3. Results

### 3.1. DnaK and TF mediate thermotolerance

**Figure 1:**
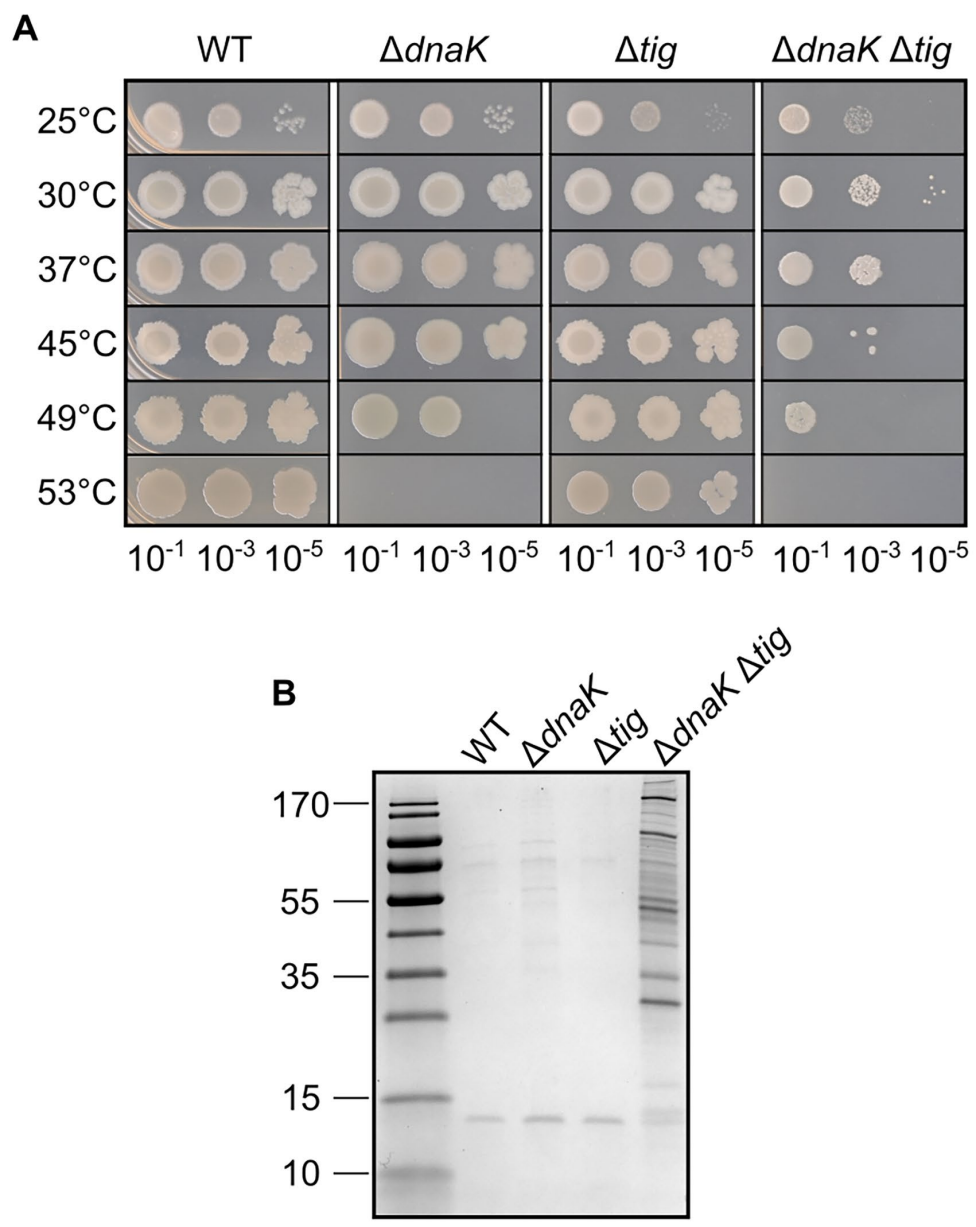
Heat tolerance and protein aggregation upon deletion of *dnaK* and *tig*. **(A)** Spot dilution assay for *B. subtilis* wild-type (1A1), Δ*dnaK* (LUW876), Δ*tig* (LUW901) and Δ*dnaK* Δ*tig* (LUW878) grown in liquid media at different temperatures. Serial dilutions of the indicated strains were spotted on TBAB. A 10^−7^ dilution was also spotted but not shown, since none of the strains grew. Plates were incubated at the indicated temperatures for 24 h. **(B)** Protein aggregates in the indicated strains grown at 30°C. Proteins were separated by SDS-PAGE and the gel stained with Coomassie blue. Molecular mass markers (10, 15, 25, 35, 40, 55, 70, 100, 130, and 170 kDa) are shown in the left lane.

We began by testing whether the absence of *dnaK* and *tig*, and the combination of both deletions, would affect the ability of the cells to grow at elevated temperatures. Serial dilutions of cultures of *B. subtilis* 1A1 (wild-type), LUW876 (Δ*dnaK*), LUW901 (Δ*tig*), and LUW878 (Δ*dnaK* and Δ*tig*) were spotted on agar plates separately, and the plates were incubated at different temperatures. Apart from a slight defect on growth at 25°C, deletion of *tig* did not affect cell viability at any of the tested temperatures (Figure 1A). Whereas the wild-type and *tig* single mutant exhibited similar viability at all temperatures tested, growth of the *dnaK* mutant strain was affected at 49°C, and absent at 53°C, confirming what has been previously reported by Schulz et al. (1995). The double *dnaK tig* mutant appeared to grow less well than the other strains at all tested temperatures. Its growth defects were far more severe with the increase in temperature, and like the *dnaK* single mutant, the double mutant did not grow at 53°C (Figure 1A). These results show that deleting *tig* in a *dnaK* mutant strain further decreases thermotolerance. In addition, we analyzed intracellular protein aggregation by SDS-PAGE. Pronounced protein aggregation in the *dnaK tig* double mutant was detected in extracts from cells grown at 30°C and 37°C (Figure 1B; Supplementary Figure S1). The respective single mutants did not seem to accumulate protein aggregates, similar to the wild-type, with the exception of the *tig* mutant at 37°C which showed a notable amount of aggregates (Supplementary Figure S1). Taken together the results suggest that the presence of both chaperones is important for cellular viability and thermotolerance but also for prevention of protein aggregation already at typical growth temperatures.

### 3.2. Twisted cell morphology and reduced colony size in cells lacking both DnaK and TF

**Figure 2:**
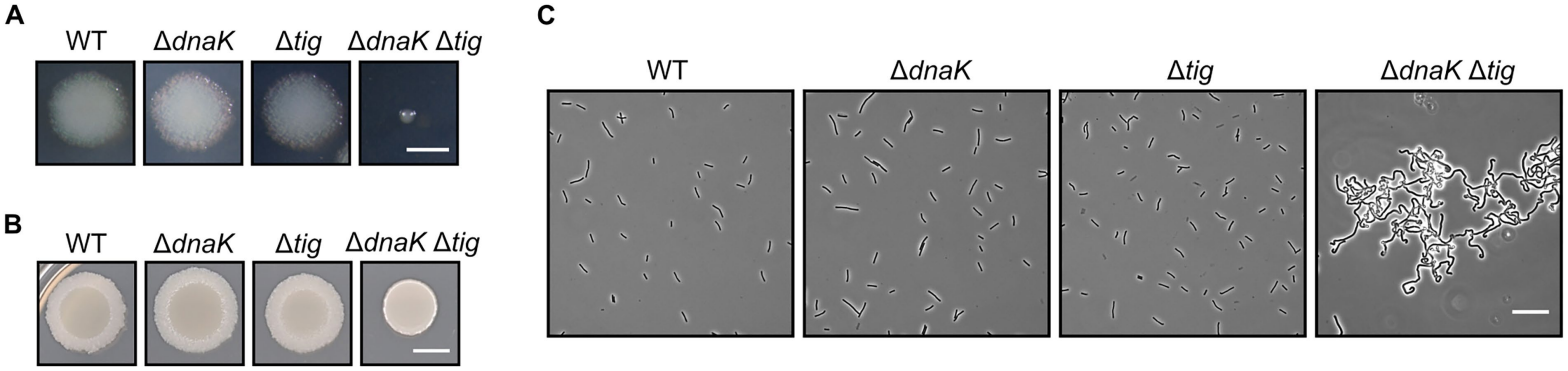
Colony size and cell morphology of chaperone deletion mutants. **(A)** Depiction of single colonies of *B. subtilis* wild-type (1A1), Δ*dnaK* (LUW876), Δ*tig* (LUW901), and Δ*dnaK* Δ*tig* (LUW878) from overnight TBAB plates incubated at 30°C. The scale bar represents 1 mm. **(B)** Shown are TBAB plates inoculated with 5-μl drops of the four indicated strains and incubated at 30°C. Cells for the drop inoculum were taken from LB cultures at mid-exponential growth phase. The scale bar represents 0.5 cm. **(C)** Representative phase contrast micrographs of the four indicated strains. Cells were taken from single colonies from overnight TBAB plates incubated at 30°C. The scale bar represents 20 μm.

**Figure 3:**
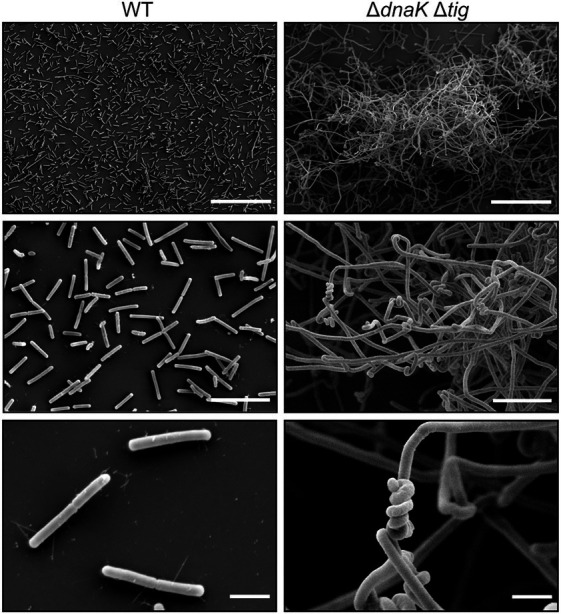
Twisted morphology of the *dnaK tig* double mutant. Representative SEM images of *B. subtilis* wild-type (1A1), on the left panels, and Δ*dnaK* Δ*tig* (LUW878), on the right panels. Scale bars represent 50 μm for the top panels, 10 μm for the middle panels, and 2 μm for the bottom panels.

We found that the *dnaK tig* double mutant displayed a much smaller colony size when compared to the wild-type (Figure 2A). In addition, when inoculating bacterial culture drops (5 μl) on TBAB plates, the *dnaK tig* double mutant did not spread on the plate to the same extent as the wild-type, or the single deletion strains (Figure 2B). To further characterize the *dnaK tig* double mutant, we observed cells from the small colonies with phase contrast microscopy and with a scanning electron microscope (SEM) noting an aberrant cell morphology (Figures 2C, 3). The *dnaK tig* double mutants displayed long chains of cells forming filaments with twists (with 96% of the cells forming filaments, and 27.5% of twists; Supplementary Table S2). No twists were observed in the wild-type or in the *dnaK* and *tig* single mutants. Filamentation was 6% in the *tig* mutant, and absent in the wild-type and *dnaK* mutant strains (Supplementary Table S2).

The SEM analysis clearly showed that the chaining cells occasionally form helical-like twists (Figure 3). Interestingly, the twisted morphology was seen in bacterial cultures incubated at 30°C and 37°C, but barely at 25°C, after an overnight incubation on TBAB plates (Supplementary Figure S2). However, twists could be observed after growing the strain for an extra day (Supplementary Figure S2) also when cultures were incubated at 25°C. The twisted morphology was observed from bacteria grown on solid media (Figure 2C) and was observed to a lesser degree when bacteria were grown in liquid cultures (NSMPG; Supplementary Figure S3).

The reduced colony size and the twisted morphology in the *dnaK tig* double mutant were also observed when using other solid growth media (NSMPG and minimal medium supplemented with glucose; Supplementary Figures S4, S5), thus making sure that the observed growth defects were not specific to TBAB. The reduced spreading of the *dnaK tig* double mutant was also seen on NSMPG plates (Supplementary Figure S4), but none of the strains managed to spread on the minimal medium plates (Supplementary Figure S5). Note that, similar to when the strain was incubated at 25°C, twists on minimal medium appeared after a longer incubation time (Supplementary Figure S5). The delayed onset of the curls in conditions where growth rate is reduced (lower temperature and minimal medium) suggests that accumulation of misfolded proteins slows down possibly due to an overall changing of the translation rate.

**Figure 4:**
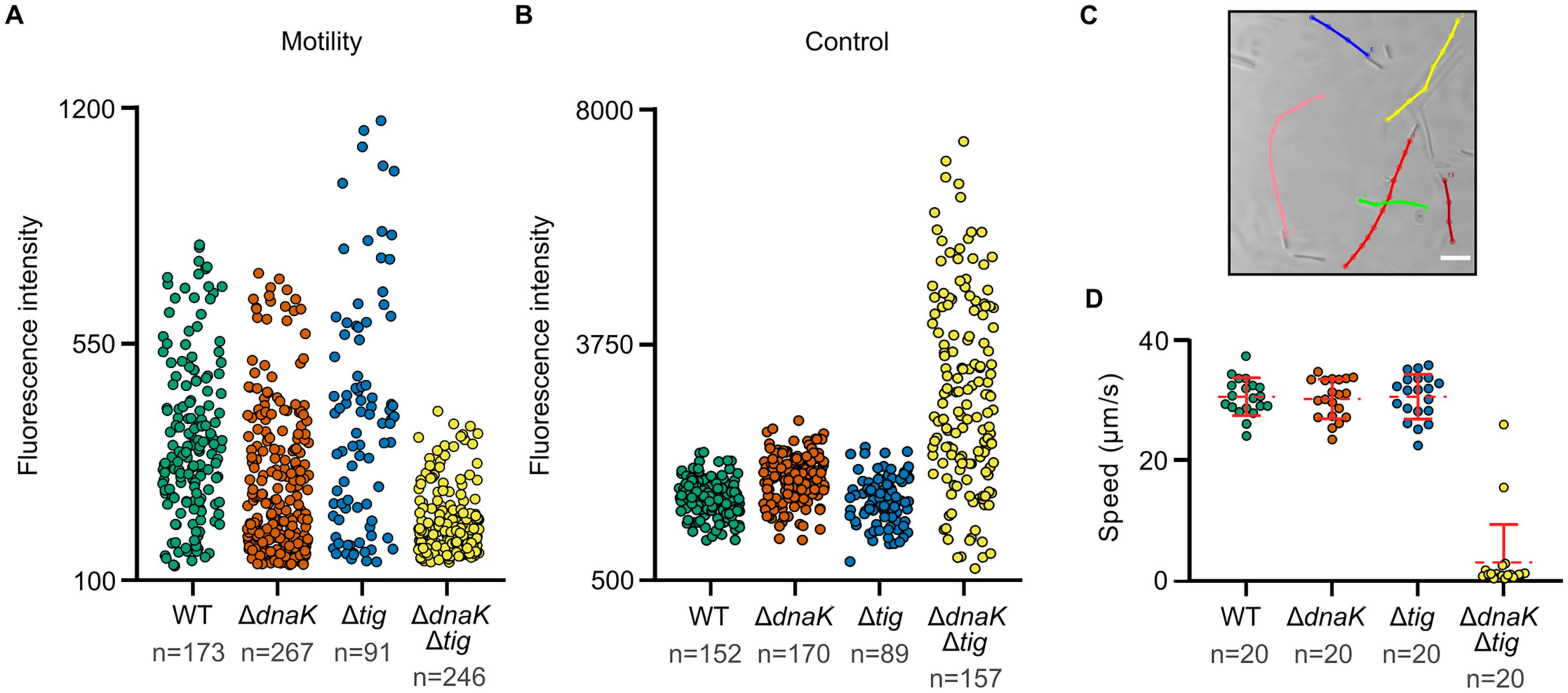
Motility of chaperone deletion mutants. **(A,B)** Fluorescence intensities of the four indicated strains expressing *P_hag_-gfp* and *P_const_-gfp*, respectively. Each dot represents the mean fluorescence intensity of a single cell. *n* denotes the number of cells analyzed for each strain. Mean intensities per cell were calculated by MicrobeJ (Ducret et al., 2016). Note that differences in motility gene expression between strains were assessed with the Mann–Whitney statistical test (two-tailed; significance level of 0.01). Pairwise comparisons were done, and the obtained *p*-values were corrected for multiple testing. Details can be found in Supplementary Data Sheet 1. Value of *p* <  0.01 were obtained for the following pairwise comparisons: wild-type and *dnaK* single mutant; wild-type and *dnaK tig* double mutant; *dnaK* single mutant and *dnaK tig* double mutant. **(C)** Representative example of *B. subtilis* wild-type (1A1) motility tracks. To study motility, microscopy videos were acquired for each strain, and individual cells were manually tracked using the MTrackJ plugin of Fiji (Schindelin et al., 2012). Cells for imaging were taken from NSMPG liquid cultures grown at 30°C until late exponential growth phase. Scale bars represent 10 μm. **(D)** Filled dots show individual speed values of the different strains. *n* denotes the number of cells analyzed for each strain. Red horizontal lines represent the mean, and errors bars display the standard deviation between speed values.

We then investigated whether the reduced colony size of the *dnaK tig* double mutant, as well as its difficulties in spreading on agar surfaces, would be linked to defects in motility. We used a GFPmut2 transcriptional reporter fusion to the promoter of flagellin (P*_hag_*-*gfp*) as a proxy for motility. Non-motile cells have a low expression of *hag.* While a subgroup of cells from the wild-type or the single deletion strains expressed high fluorescent values, none of the analyzed *dnaK tig* double mutant cells exhibited fluorescent values in this higher range, indicating a reduced motility gene expression (Figure 4A; Supplementary Figure S6). We subjected the fluorescence intensity data to the Mann–Whitney statistical test (two-tailed; significance level of 0.01). An obtained value of *p* < 0.01 indicated that the observed differences in motility gene expression between the *dnaK tig* double mutant and the wild-type strains were statistically significant. Differences in fluorescence intensity values between the wild-type and the *tig* mutant were not found to be significant (value of *p* > 0.01). Note that absence of *dnaK* alone negatively affected motility (value of *p* < 0.01), but that a significant reduction in motility was observed in the double mutant when compared to the *dnaK* population (value of *p* < 0.01). Details regarding the performed Mann–Whitney test can be found in Supplementary Data Sheet 1. To verify that low intensity fluorescence values in the double mutant were not due to a general problem affecting folding of the GFPmut2-based fluorescence reporter, we used a strain in which expression of the gene encoding GFPmut2 was driven by a constitutive promoter (Figure 4B; Supplementary Figure S6). Wild-type, *dnaK* and *tig* single mutant strains showed a small cell to cell variation in GFPmut2 fluorescence intensity in contrast to the large variability seen for the *dnaK tig* double mutant cells (Figure 4B). The aberrant cell morphology and possible fluctuations in the amount or activity of molecules related to transcription and translation in the cells that lack of the two chaperones may explain the observed variation. We also tracked the movement of single cells in real time under the microscope. While the wild-type and *dnaK* and *tig* single mutant strains moved at a similar speed, the *dnaK tig* double mutant cells showed a reduction in motility (Figures 4C,D).

Subsequent complementation experiments showed that colony size, spreading on solid surfaces, and the aberrant morphology could be restored by an ectopically integrated copy of the *tig* gene under control of its native promoter at the *amyE* locus (Supplementary Figure S7). We also assessed whether complementation would occur when expressing *tig* from an IPTG-inducible promoter. Without IPTG induction, we observed a slight increase in colony size and a partial morphology restoration, suggesting that small amounts of *tig* expression (due to the leakage from the P*_hyperspank_* promoter) partially complemented the *dnaK tig* double mutant (Supplementary Figure S8). Higher *tig* expression (achieved by adding 1 mM IPTG to TBAB plates) greatly improved the phenotype of the double mutant, but did not lead to full complementation as seen with the native promoter (Supplementary Figures S7, S8). This indicates that the levels and/or timing of *tig* expression from the P*_hyperspank_* promoter are not sufficient for full colony size and morphology restoration. Similarly, a slight increase in colony size and partial morphology restoration was observed when complementing the *dnaK tig* double mutant with expressed *dnaK* from an IPTG-inducible promoter. The morphology restoration was more clearly observed after an extended incubation time (Supplementary Figure S9). Whole-genome sequencing of LUW878 (Δ*dnaK* Δ*tig*) confirmed that it did not contain additional mutations apart from *dnaK* and *tig* deletions. Taken together, the absence of both DnaK and TF leads to a pleiotropic phenotype that is not observed for the respective single gene mutants.

### 3.3. Cell division, growth and sporulation in the absence of DnaK and TF

**Figure 5:**
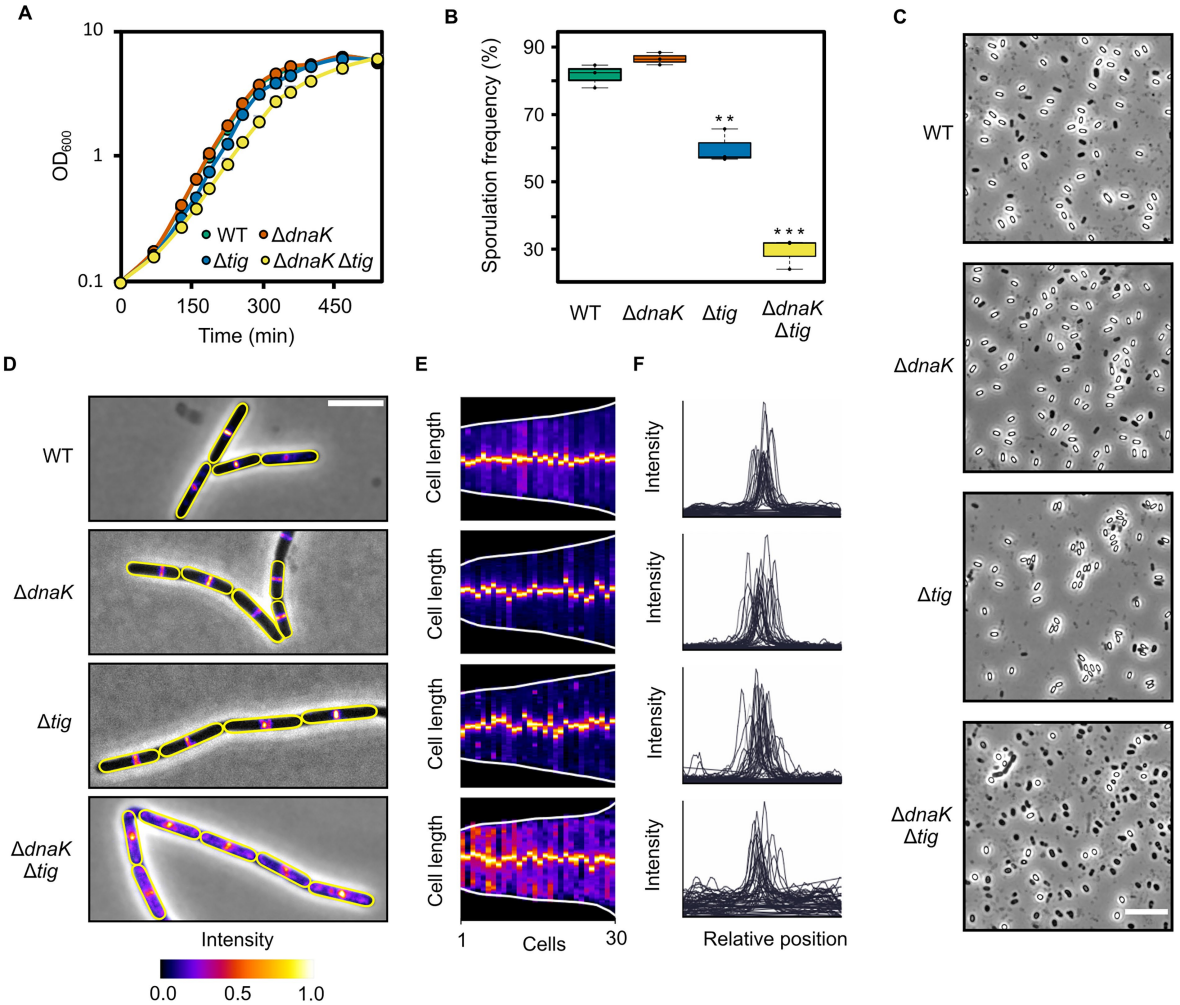
Growth, sporulation, and cell division of chaperone deletion mutants. **(A)** Growth curves of *B. subtilis* wild-type (1A1), Δ*dnaK* (LUW876), Δ*tig* (LUW901), and Δ*dnaK* Δ*tig* (LUW878). Cells were grown in a liquid broth medium (NSMPG) at 30°C. Each strain was grown in triplicates, and represented are the average OD_600_ measurements. Error bars displaying the standard deviation between the triplicates are also represented, but due to the small variation, they are not seen. **(B)** Sporulation frequencies of the four indicated strains, which were grown in NSMP liquid broth medium at 30°C for 48 h. Error bars display the standard deviations for the triplicates. The values were subjected to a two-tailed homoscedastic *t*-test, comparing each strain with the wild-type (1A1). Significant differences are denoted by two asterisks (value of *p* < 0.01) or three asterisks (value of *p* < 0.001). **(C)** Phase contrast micrographs showing spores of indicated strains. Cells were incubated on NSMP plates for ten days at 30°C before imaging. The scale bar represents 10 μm. **(D)** Micrographs of the indicated strains expressing mNG-ZapA to visualize the Z-ring. Shown are overlays of the phase contrast and fluorescence channel (mNG). Cells were grown until mid-exponential growth phase in NSMPG at 30°C. Cell outlines are in yellow. Scale bar represents 5 μm. **(E)** Demographs of the indicated strains displaying the mNG-ZapA fluorescence intensity over the cell length axis. Each slice represents one cell, and cells were sorted by length from shortest to longest. Thirty cells were analyzed for each strain. Demographs were generated by MicrobeJ (Ducret et al., 2016). **(F)** Fluorescence intensity profiles over the normalized cell length of the indicated strains. For each strain, intensity profiles of 30 cells were plotted. Graphs were generated by MicrobeJ (Ducret et al., 2016).

Even though morphology and colony size were severely affected in the double mutant, cells were able to grow in liquid cultures with no major impediments but just a slightly longer doubling time (Figure 5A). The generation time for the double mutant was 57.8 ± 1.0 min, while it was 49.1 ± 1.2, 48.0 ± 0.4, and 47.0 ± 0.2 min for the wild-type and *dnaK* and *tig* single mutants, respectively.

Next, we sought to investigate if sporulation, one of the most well-characterized cellular differentiation processes in bacteria, would be affected by the double *dnaK tig* deletion. In NSMP liquid cultures, the double mutant was capable of asymmetrically dividing into spores, although with a lower frequency (29.2 ± 3.7%) than the wild-type strain (81.6 ± 2.8%) (Figure 5B; Supplementary Table S3). The sporulation frequencies for the *dnaK* and *tig* single mutants were 87.4 ± 2.8% and 59.9 ± 4.1%, respectively (Figure 5B; Supplementary Table S2). On NSMP agar plates, spores were observed for the *dnaK tig* double mutant, but also at a much lower frequency (17.0% ± 1.7%) than the wild-type strain (74.0 ± 2.7%) (Figure 5C; Supplementary Table S4). The proportion of spores in the *dnaK* and *tig* single mutant population was 63.9 ± 3.9% and 80.6 ± 2.5%, respectively. These data shows that even though the sporulation frequency is clearly affected by the double *dnaK tig* mutation, some cells manage to form spores. This is in stark contrast to, for example, a sporulation-defective *spo0A* mutant, for which almost no spores are formed (Winstedt and von Wachenfeldt, 2000). Finally, we investigated whether cell division is affected in the *dnaK tig* double mutant strain. Cell division is governed by FtsZ, which self-assembles into protofilaments and forms the contraction ring (Z-ring) (Bi and Lutkenhaus, 1991; Dajkovic and Lutkenhaus, 2006). We explored if Z-rings were formed and localized normally using mNeonGreen-ZapA (mNG-ZapA) fusion protein as a proxy for assembly of native FtsZ rings. ZapA is an FtsZ-associated protein (Gueiros-Filho and Losick, 2002), and thus allows visualization of Z-rings under the fluorescence microscope when fused to the fluorescent protein mNG. We did not find any major defects in Z-ring formation in the *dnaK tig* single mutants, as the peak fluorescence signal formed at mid-cell positions (Figures 5D–F), similar to what was observed in the wild-type strain. However, we noted that at several filament ends that seemed aberrant, mNG-ZapA appeared de-localized (Supplementary Figure S10). We then visualized the localization pattern of MreB, which is majorly associated with control of the rod shape (Doi et al., 1988; Levin et al., 1992). We observed no apparent major differences in the MreB localization pattern of the *dnaK* and *tig* single and double mutant strains when compared to the wild-type, with the exception of damaged filament ends which showed abnormal fluorescence intensities and patterns (Supplementary Figure S11). We surmise that the *dnaK tig* double mutant grows in very long filaments that often break due to physical shear forces.

Overall, these data indicate that, despite the pleiotropic phenotype of the *dnaK tig* double mutant – which includes a filamentous twisted morphology – cell growth and cell division do not appear to be largely compromised, and sporulation is only moderately affected.

### 3.4. Cell wall integrity is decreased in the *dnaK tig* double mutant

**Figure 6:**
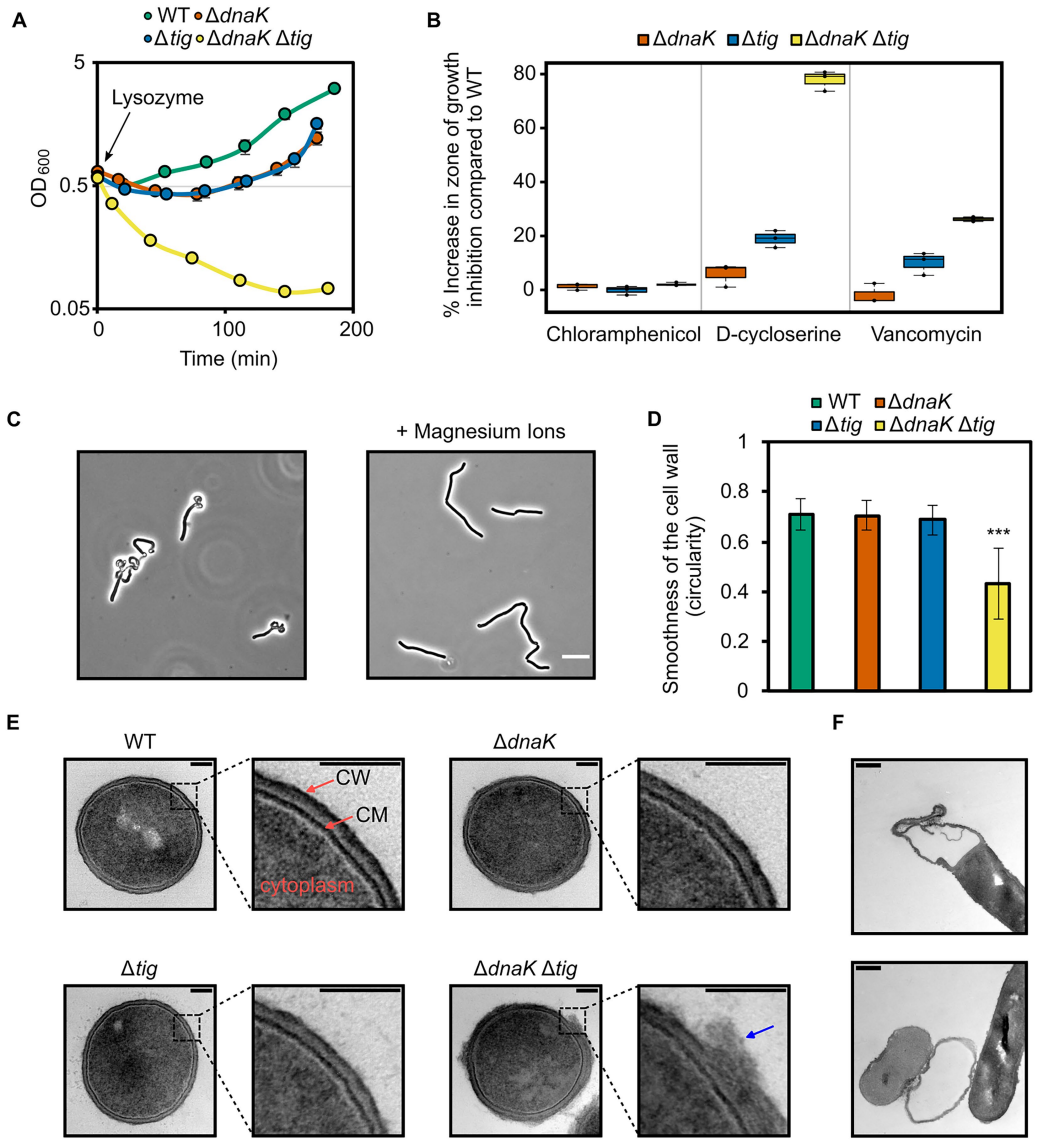
Effects of *dnaK* and *tig* deletions on cell wall integrity. **(A)** Cell density measured by OD at 600 nm of *B. subtilis* wild-type (1A1), Δ*dnaK* (LUW876), Δ*tig* (LUW901), and Δ*dnaK* Δ*tig* (LUW878) strains after the addition of 15 μg/ml lysozyme at OD_600_ = 0.6 (*t* = 0). Strains were grown in NSMPG at 30°C. Each strain was grown in triplicates, and error bars represent the standard deviation between them. Error bars might not be seen due to the small variation. **(B)** Sensitivity of the indicated strains to chloramphenicol (protein synthesis inhibitor), D-cycloserine and vancomycin (antibiotics targeting the cell wall). Plotted is the increase in percentage in the zone of growth inhibition when compared to the wild-type strain. Three antibiotic discs were used for each strain, and error bars display the standard deviation between the triplicates. Plates with antibiotic discs were incubated at 30°C for 20 h. **(C)** Representative phase contrast micrographs of Δ*dnaK* Δ*tig* cells from overnight TBAB plates without (left image) or supplemented with 25 mM MgSO_4_ (right image). Plates were incubated at 30°C. Scale bar represents 10 μm. **(D)** Smoothness of the cell wall of the four indicated strains. Smoothness was determined by measuring circularity values of the cell wall outlines in Fiji (Schindelin et al., 2012). TEM transversal sections from 10 cells (as the ones shown in “E”) were analyzed per each strain. Error bars display the standard deviations for the triplicates. The values were subjected to a two-tailed heteroscedastic t-test, comparing each strain with the wild-type (1A1). Significant differences are denoted by asterisks (***: value of *p* < 0.001). **(E)** Representative TEM transversal sections of the indicated strains. Cells were grown until exponential phase in a liquid broth medium (NSMPG) at 30°C. Scale bars represent 100 nm. “CM”: cytoplasmic membrane; “CW”: cell wall. Blue arrow indicates cell wall material that appeared loose from the surface. **(F)** TEM longitudinal sections showing Δ*dnaK* Δ*tig* (LUW878) “empty” cells. Scale bars represent 100 nm.

The cell wall is the major determinant of cell shape in bacteria (Cabeen and Jacobs-Wagner, 2005). Since the *dnaK tig* double mutant exhibited a twisted cell shape, we investigated whether the strain had defects in the structural and functional integrity of the cell wall. We began by growing the double mutant until mid-exponential growth phase after which the peptidoglycan hydrolase lysozyme was added. The wild-type, *dnaK*, and *tig* single mutant strains continued growing after a minor drop in cell density (Figure 6A). However, the *dnaK tig* double mutant displayed a large drop in cell density and could not recover from the exposure to lysozyme (Figure 6A). We also tested the strains for sensitivity to D-cycloserine and vancomycin, which are antibiotics that inhibit bacterial cell wall synthesis. Vancomycin binds to the D-Ala-D-Ala C-terminus of peptidoglycan precursors, inhibiting cell wall synthesis (Courvalin, 2006), and D-cycloserine blocks cell wall synthesis by inhibiting two sequential enzymes involved in the formation of the D-Ala-D-Ala dipeptide (Neuhaus and Lynch, 1964). As seen in Figure 6B, sensitivity to both antibiotics (when bacteria were grown on TBAB plates) was increased in the *dnaK tig* double deletion strain. When compared to the wild-type strain, the zone of growth inhibition for the *dnaK tig* mutant with D-cycloserine and vancomycin increased by 77.9 ± 3.0% and 26.2 ± 0.7%, respectively. These numbers were 18.9 ± 2.6% and 10.1 ± 3.4% for the *tig* mutant - indicating that the *tig* mutant is also affected by cell wall antibiotics, but to a lesser extent compared to the double mutant, and a 5.9 ± 3.4% increase and a 1.8 ± 3.0% decrease for the *dnaK* mutant. As a control, we also tested the four strains for sensitivity to chloramphenicol, since this antibiotic does not target the cell wall, but inhibits protein synthesis by binding to the 50S ribosomal subunit (Thompson et al., 2002). When compared to the wild-type, the zones of growth inhibition marginally increased by 1.3 ± 1% and 2.1 ± 0.5% for the *dnaK* and *dnaK tig* mutant strains, and remained the same for the *tig* mutant (−0.1 ± 1.3%; Figure 6B). Sensitivities to both antibiotics were also increased in the *dnaK tig* double mutant when grown in liquid medium, as determined by minimal inhibitory concentration (MIC) assays (Supplementary Table S5).

Millimolar concentrations of magnesium ions have been shown to partially restore aberrant morphologies that deviate from the typical rod-shaped *B. subtilis* (Murray et al., 1998; Formstone and Errington, 2005; Schirner and Errington, 2009b; Mehne et al., 2013; Sassine et al., 2020; Tesson et al., 2022), and Mg^2+^ rigidifies the cell wall by inhibiting autolysis, shifting the balance between cell wall synthesis and degradation towards synthesis of new cell wall material (Dajkovic et al., 2017; Tesson et al., 2022). When the *dnaK tig* double mutant was grown on TBAB plates with added magnesium, the aberrant morphology was partially restored (Figure 6C).

To directly observe the cell wall at high resolution, we imaged exponentially growing cells by transmission electron microscopy (TEM). We found that the *dnaK tig* double mutant exhibited a rough and irregular cell wall surface, with outer cell wall material that appeared loose from the surface. In stark contrast to these observations, wild-type and *dnaK* and *tig* single mutant cell walls were smooth and mostly homogenous (Figures 6D,E). We also noted many “ghost” cells in the double mutant strain (Figure 6F), that presumably leaked out their cytoplasmic contents due to a damaged cell wall. These “ghost” cells were not observed in the TEM images from the wild-type or the *dnaK* and *tig* single mutants. Overall, these results indicate that cell wall integrity is markedly affected by the *dnaK tig* double deletion, and suggest that the aberrant twisted morphology is linked to a compromised cell wall integrity.

### 3.5. Spontaneous suppressor mutations in the *dnaK tig* double mutant partially suppress the twisted morphology

**Figure 7:**
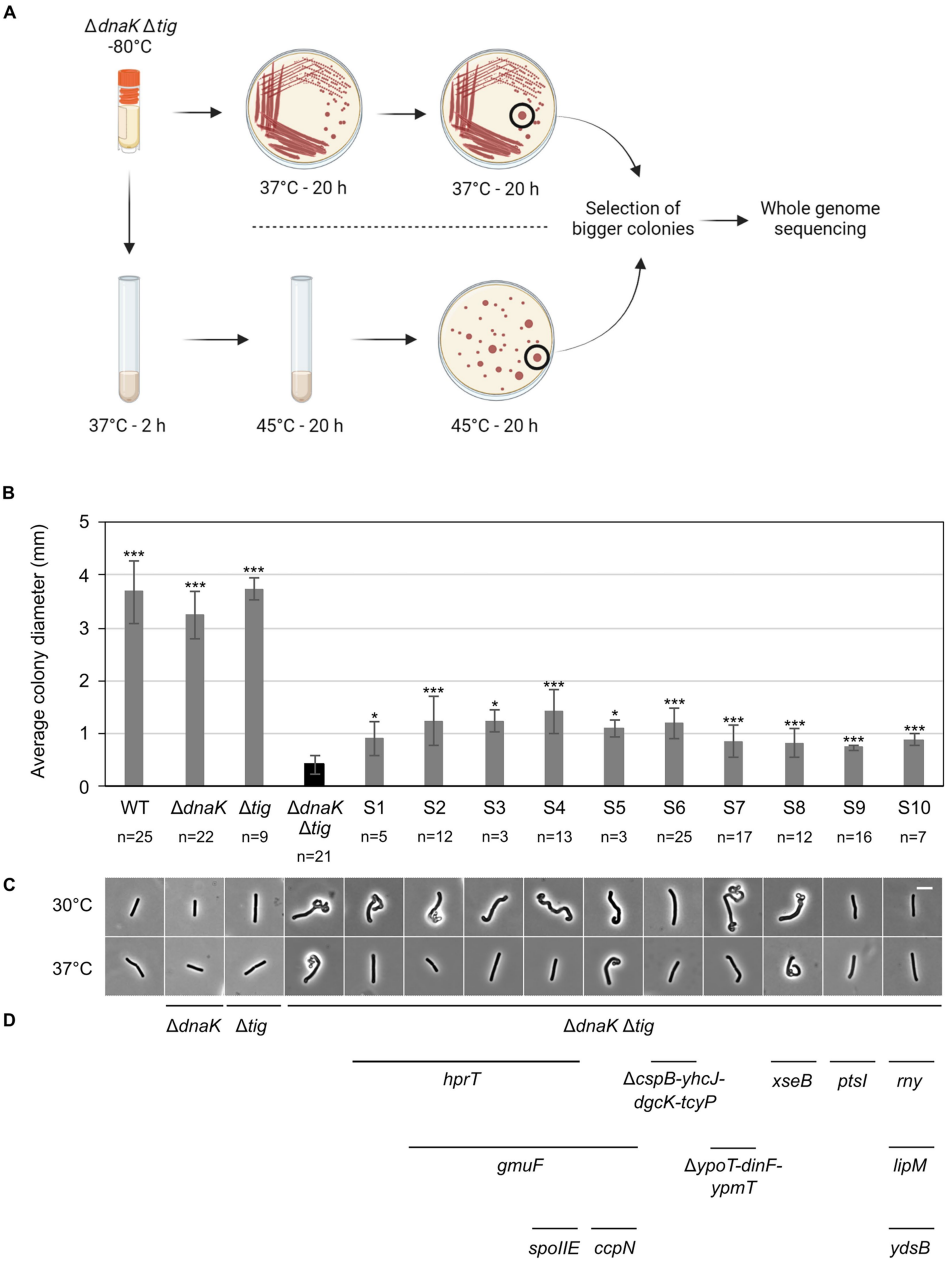
Isolation of LUW878 (Δ*dnaK* Δ*tig*) suppressors with partially restored colony size and cell morphology. **(A)** Isolation of LUW878 suppressors was achieved by two methodological approaches. In the first approach, the strain was streaked for single colonies from a glycerol stock stored at −80°C onto a TBAB plate. After an overnight incubation at 37°C, cells were re-streaked and incubated again at the same temperature. Suppressor mutants were isolated from the plates by selecting colonies that were larger than LUW878 itself. In a second approach, LUW878 was grown in 2 ml LB liquid medium at 37°C for 2 h, and then shifted to 45°C for another 20 h. Bacteria in different dilutions were plated on TBAB and grown at 45°C overnight. Suppressor mutants were isolated by selecting larger colonies. In total, ten suppressors were isolated and whole-genome sequenced. **(B)** Average colony diameters of the ten selected Δ*dnaK* Δ*tig* suppressors (S1–S10). *n* denotes the number of colonies on each plate. Colony diameters were measured with Fiji (Schindelin et al., 2012). Asterisks indicate *p*-values from two-tailed heteroscedastic *t*-tests with the null hypothesis Østrain = ØLUW878; *: value of *p* < 0.05; ***: *p* < 0.001. **(C)** Representative phase contrast micrographs of the isolated suppressors. Cells for imaging were taken from single colonies present on overnight TBAB plates incubated at 30°C or 37°C. The scale bar represents 5 μm. **(D)** Genes, identified through whole-genome sequencing, that either where deleted or contained point mutations are indicated below the micrographs of the respective suppressor strain.

To gain further insight into which biological processes were affected in the *dnaK tig* mutant cells, we screened for second-site suppressor mutants. Larger colonies occasionally appeared after re-streaking the double mutant and incubating it at 37°C (but not at 25°C or 30°C). The fact that suppressor mutants with increased colony size were easily obtainable at 37°C goes in line with our previous observations showing that (I) at 37°C, cells not only appeared twisted, but also showed signs of lysis that were absent at lower temperatures (Supplementary Figure S1), and (II) the strain lacking DnaK and TF was sensitive to elevated temperatures (Figure 1A). We picked and kept isolates of eight colonies with increased size (designated suppressor S1 to S8) (Figure 7A). As another strategy to select potential suppressors, we grew the cells at 37°C, shifted them to 45°C, and picked two colonies with increased size (designated S9 and S10). In addition to a slightly increased colony size (Figure 7B), most suppressors partially restored the twisted morphology (Figure 7C; Supplementary Figure S12). On some occasions, the morphological restoration appeared to be conditional to the temperature, with cells that were mostly straight at 37°C, but still twisted at 30°C (Figure 7C; Supplementary Figure S12). Chromosomal DNA from the ten isolated clones (S1 to S10) was extracted and subjected to whole-genome sequencing. We identified mutations in several genes (Table 3; Figure 7D) that are involved in diverse biological processes, including carbon metabolism (*ccpN, gmuF,* and *ptsI*), nucleotide metabolism (*hprT* and *dgcK*), RNA synthesis and degradation (*cspB* and *rny*). Some of the identified genes were involved in regulating gene expression (*hprT*, *spoIIE*, *ccpN*, *ypoP*, *ysdB*), and half the genes encode for membrane proteins (*spoIIE*, *yhcJ*, *dgcK*, *tcyP*, *dinF*, *ypmT*, *rny*, and *ysdB*). Additional description of the identified genes is summarized in Supplementary Table S3. Taken together, these data suggest that *B. subtilis* can adjust several cellular processes to improve colony size and cell morphology defects caused by the lack of DnaK and TF.

**Table 3:** Second-site suppressor mutations in *dnaK tig* double mutant strains.

Gene	Coding region change^a^	Strain(s)	Type	Gene category^b^				
				1	2	3	4	5
*hprT*	A44G (E15G)	S1	Metabolic enzyme, transcription factor					
	T194C (M65T)	S2, S3, S4						
*gmuF*	909_910insT (I315fs)	S2, S3, S4, S5	Metabolic enzyme					
*spoIIE*	C2156T (P719L)	S4	Phosphatase					
*xseB*	20delA (N7fs)	S8	Exonuclease					
*ccpN*	T371C (L124P)	S5	Transcription factor					
*cspB*	Δ*cspB*	S6	RNA chaperone					
*yhcJ*	Δ*yhcJ*	S6	ABC transporter					
*dgcK*	Δ*dgcK*	S6	Diguanylate cyclase					
*tcyP*	Δ*tcyP* (0_1195del)	S6	Transporter					
*ypoP*	Δ*ypoP* (K77*)	S7	Transcription factor					
*dinF*	Δ*dinF*	S7	Na^+^-driven efflux pump					
*ypmT*	Δ*ypmT* (Y41fs)	S7	Unknown membrane protein					
*ptsI*	G553C (G185R)	S9	Phosphotransferase system enzyme I					
*rny*	637_638delinsAA (V213N)	S10	Rnase Y					
	G940A (G314S)	S10						
*lipM*	C110A (P37Q)	S10	Octanoyltransferase					
*ysdB*	A258G	S10	SigW pathway protein					

^a^Deletion (del), insertion (ins), frame shift (fs), non-sense mutation (*). ^b^Gene categories as assigned in SubtiWiki (Zhu and Stulke, 2018): 1, Metabolism; 2, Coping with stress; 3, Regulation of gene expression; 4, Membrane protein; 5, DNA/RNA synthesis or degradation.

### 3.6. Properties of suppressors with restored morphology

**Figure 8:**
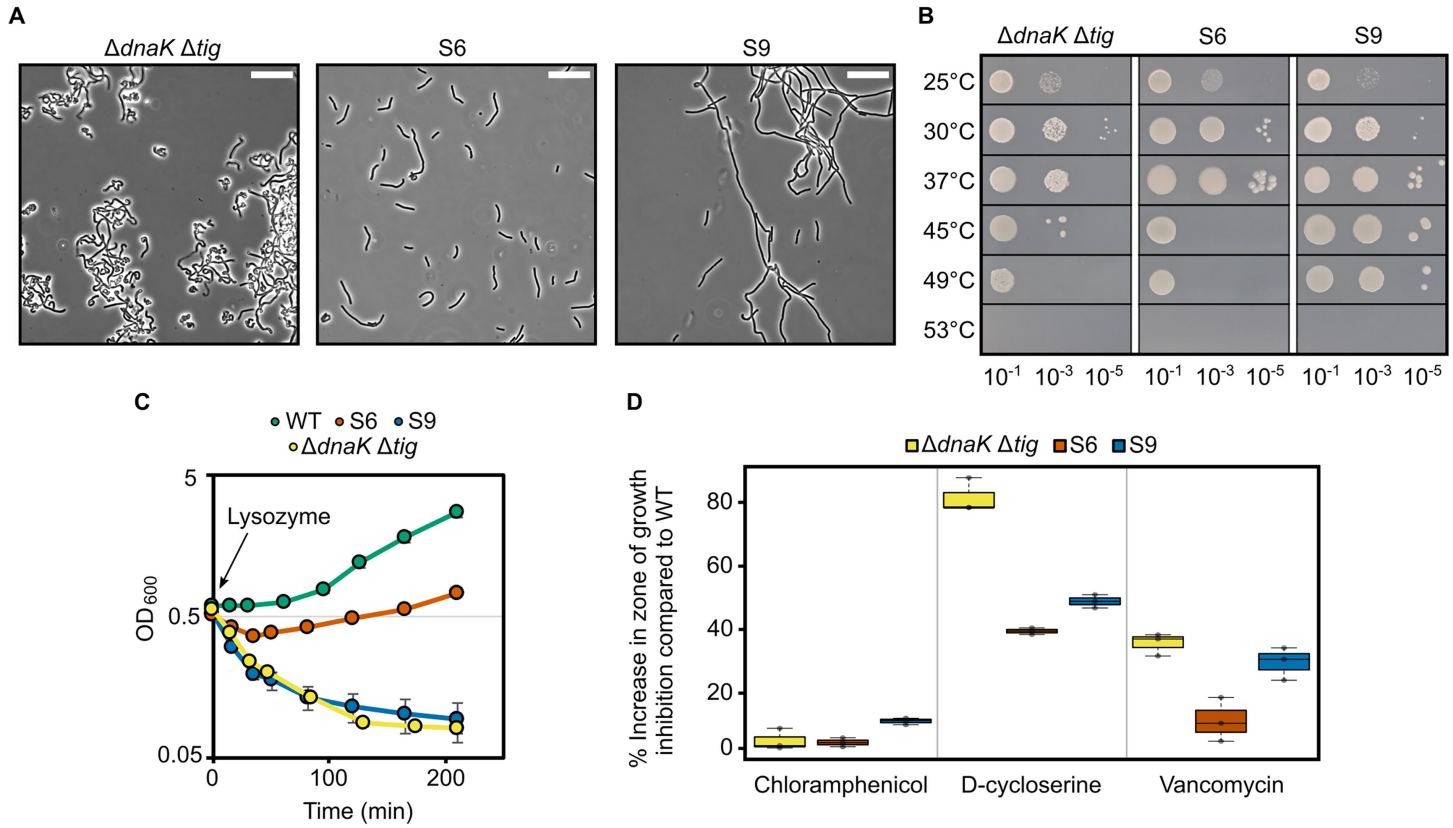
Properties of two selected suppressors with a partially restored morphology. **(A)** Phase contrast micrographs of *B. subtilis* Δ*dnaK* Δ*tig* (LUW878) and the LUW878 suppressors S6 and S9. Cells were taken from single colonies from overnight TBAB plates incubated at 30°C. The scale bar represents 20 μm. **(B)** Spot dilution assay for the indicated strains at different temperatures. Serial dilutions were spotted on TBAB plates. 10^−7^ dilution was also spotted but not shown, since none of the strains grew. Plates were incubated at the indicated temperatures for 24 h. **(C)** Cell density measured by OD at 600 nm of the indicated strains after the addition of 15 μg/ml lysozyme at OD_600_ = 0.6 (*t* = 0). Strains were grown in NSMPG at 30°C. Each strain was grown in triplicates, and error bars represent the standard deviation between them. Error bars might not be seen due to the small variation. **(D)** Sensitivity of the indicated strains to chloramphenicol (protein synthesis inhibitor), D-cycloserine and vancomycin (antibiotics that target the cell wall). Plotted is the increase in percentage in the zone of growth inhibition when compared to the wild-type strain. Plates with the antibiotic discs were incubated at 30°C for 20 h. Three antibiotic discs were used for each strain, and error bars display the standard deviation between the triplicates.

To determine if morphology restoration was accompanied by restoration of other phenotypic properties, we selected suppressors S6 and S9 and tested their heat tolerance and sensitivity to D-cycloserine, vancomycin, and lysozyme. S6 and S9 were selected out of the ten suppressors because (I) they showed partial morphology restoration both at 30°C and 37°C (Figures 7C, 8A; Supplementary Figure S12), (II) were isolated with two different methodological approaches, and (III) contained only one mutation (a deletion and a single point mutation, respectively; Table 3). S6, isolated at 37°C with the first approach (Figure 7A), did not show any improved tolerance to temperatures above 37°C (Figure 8B). In contrast, S9, which was isolated at 45°C with the second approach (Figure 7A), did display improved heat tolerance, as evident by increased cell survival at 45°C and 49°C (Figure 8B). Both S6 and S9 were more tolerant to D-cycloserine. S6, but not S9, also showed less sensitivity to lysozyme and to vancomycin, suggesting it had improved cell wall integrity (Figures 8C,D). These results suggest that, apart from morphology, other phenotypic traits of the *dnaK tig* double deletion strain can be partially suppressed.

## 4. Discussion

Cells employ a number of protein folding and degradation systems to maintain proteostasis. Key to these processes in most bacteria are the ubiquitous molecular chaperones DnaK and TF (Hartl and Hayer-Hartl, 2002). In some bacteria DnaK is essential for growth under both stress and non-stress conditions and may perform additional functions not directly related to its folding activity (Schramm et al., 2017). In *E. coli* and *B. subtilis* DnaK and TF are dispensable for cell growth in the absence of stress due to partially overlapping functions and redundancy with other chaperone systems (Deuerling et al., 1999; Teter et al., 1999; Deuerling et al., 2003; Genevaux et al., 2004; Vorderwulbecke et al., 2004; Calloni et al., 2012). The combined loss of DnaK and TF in *E. coli* leads to proteostasis collapse characterized by significantly increased levels of aggregated proteins and confers synthetic lethality at normal growth temperatures (Deuerling et al., 1999).

Here, we investigated the physiological effects of removing DnaK and TF in *B. subtilis*. We report that the *dnaK tig* double mutant has reduced tolerance to elevated temperatures and is not viable at ≥53°C. Thus, and in sharp contrast to *E. coli*, DnaK and TF are not required for viability at normal growth temperatures. We show that the combined loss of DnaK and TF is accompanied by pleiotropic changes such as a significantly reduced colony size, filamentous and twisted cell morphology and decreased tolerance to cell wall active compounds. A filamentous cell morphology is also seen in *E. coli* when DnaK or TF is either depleted or overexpressed (Paek and Walker, 1987; Bukau and Walker, 1989; Guthrie and Wickner, 1990; Blum et al., 1992) but only when both proteins are absent in *B. subtilis*. Apart from the apparent defects observed, it is striking how the lack of two major and conserved chaperones in *B. subtilis* still allows important and complex cellular processes such as cell growth, division, and spore formation to take place at the population level. The twisted, aberrant morphology in cells devoid of DnaK and TF was noted in particular when cells were grown on solid media. These cells produced very small colonies which may be explained by reduced expression of the *hag* gene encoding flagellin. The *hag* gene is expressed by RNA polymerase with the alternative sigma factor σ^D^ (Kearns and Losick, 2003) that controls a large regulon of >100 genes. The genes, *lytC*, *lytD*, and *lytF* encoding peptidoglycan-remodeling autolysins are part of the σ^D^ regulon (Chen et al., 2009). The autolysins are required for daughter cell separation and cells lacking σ^D^ grow as long filaments (Marquez et al., 1990). Thus, the observed non-motile chains of the double mutant could be due to a non-functional or less active σ^D^. However, the twisted morphology is not observed for a σ^D^ mutant. Twisted morphologies have been previously observed in *B. subtilis*, mostly in cells lacking components of the cell wall machinery (Shohayeb and Chopra, 1987; Murray et al., 1998; Jones et al., 2001; Kawai et al., 2009; Schirner et al., 2009; Schirner and Errington, 2009a; Ramaniuk et al., 2018), indicating that cell shape defects are tightly linked to cell wall homeostasis disruption. Examples of mutants with twisted shapes include cells lacking both the actin homologue MreB and the penicillin-binding protein 1 (PBP1) (Kawai et al., 2009), which are majorly involved in cell wall synthesis, and cells lacking the MreB-like protein Mbl (Jones et al., 2001; Schirner et al., 2009; Schirner and Errington, 2009a).

The bacterial cell wall, with peptidoglycan (PG) as its primary component, not only provides cells with mechanical integrity, allowing them to tolerate the internal turgor pressure, but is also a principal determinant of cell shape. Cell wall growth is mainly enabled by PG synthases, which produce and insert new cell wall material, and PG hydrolases, which cleave existing bonds so new material can be incorporated as the cell elongates (Singh et al., 2012; Sassine et al., 2020). It has been proposed that maintaining a rod-shape strongly depends on the balance between PG synthesis and hydrolysis (Singh et al., 2012; Sassine et al., 2020; Tesson et al., 2022). In line with this, removal of a glucosyltransferase involved in the teichoic acid glycolipids pathway of the cell wall in *B. subtilis* (UgtP) affects both PG synthesis and hydrolysis, but not the balance between the two, and cells appear rod-shaped. However, deleting the PG hydrolase LytE in an *ugtP* mutant creates an aberrant morphology, which can be restored not only by addition of magnesium ions (Mg^2+^), but also by expressing the PG hydrolase CwlO, or decreasing the levels of the PG synthase PBP1 (Sassine et al., 2020).

Magnesium has been shown to affect cell wall homeostasis, as addition of millimolar concentrations of magnesium ions restore the morphology of numerous aberrant-shaped mutants (Murray et al., 1998; Kawai et al., 2009; Schirner and Errington, 2009a; Mehne et al., 2013; Sassine et al., 2020), including the *dnaK tig* double mutant. Recent studies demonstrate that magnesium improves cell wall integrity by inhibiting PG hydrolases, restoring the balance between cell wall synthesis and degradation (Dajkovic et al., 2017; Tesson et al., 2022). This, together with the decreased tolerance to lysozyme and cell wall antibiotics of cells lacking both DnaK and TF, strongly suggests that the activity of PG synthases and hydrolases is dysregulated, resulting in the observed twisted cell morphology. The twisted areas of the *dnaK tig* double mutant could be zones in which PG hydrolysis prevails, with easier access of cell wall active compounds. It would be challenging, however, to identify the specific cell wall homeostasis components whose activities or functions are affected, especially because there exist more than 40 known or putative PG hydrolases in *B. subtilis* (Smith et al., 1996, 2000).

Aberrant cell morphology often appears to be conditional to temperature. For instance, mutants with altered PBP2b or PBP3 form bends and twists at 41°C, but to a much lesser extent at 37°C (Shohayeb and Chopra, 1987). Similarly, cells lacking the alternative sigma factor σ^I^ and its anti-sigma factor RsgI (Δ*sigI-rsgI* cells) display twists at 52°C, but not at 37°C (Ramaniuk et al., 2018). Interestingly, the effect of heat seems to create similar morphological abnormalities, since wild-type *B. subtilis* forms twists when grown at 50°C (Schirner and Errington, 2009a). Elevated temperature might, directly or indirectly, inactivate enzymes involved in cell wall homeostasis, leading to disrupted cell shape. In support of this, *lytE* and *mreBH* (encoding for a PG hydrolase and an MreB paralogue, respectively) were found to be strongly downregulated in a Δ*sigI-rsgI* mutant strain grown at 52°C, as well as *pdaC* encoding peptidoglycan deacetylase C involved in cell wall homeostasis (Ramaniuk et al., 2018). Similar to elevated temperature, removal of chaperones might also affect stability or proper folding of cell wall related enzymes at normal growth temperatures, leading to the loss of rod shape. In addition, chaperones may play direct roles in cell wall integrity indicated by that DnaK and GroEL chaperones are recruited to the *B. subtilis* membrane upon the addition of ethanol, contributing to stress adaptation and membrane restoration (Seydlová et al., 2012).

Spontaneous suppressor mutants from cells lacking both DnaK and TF were isolated. Most of them displayed a partial restoration of the wild-type morphology. The identified mutations in the *dnaK tig* suppressors are not in genes currently known to be directly involved in the cell wall apparatus. Instead, mutations were found in genes involved in metabolism or in other diverse cellular processes (regulation of gene expression, RNA synthesis or degradation, and coping with stress) and such mutations were sufficient to partially restore the morphology. For instance, morphology, as well as thermotolerance and cell wall integrity of the *dnaK tig* double mutant could be improved by a single missense mutation in the *ptsI* gene, encoding for the phosphotransferase system (PTS) enzyme I. Interestingly, a few studies have reported a link between cell shape regulation and metabolism in *B. subtilis*. Cells lacking MreB, which is a major component of the PG synthesis machinery, exhibit growth defects and an aberrant morphology with bulging poles (Defeu Soufo and Graumann, 2005; Formstone and Errington, 2005), and *mreB* suppressors have been identified in genes involved in metabolic processes such as *ptsI* and the carbon catabolite control protein A encoding gene *ccpA* (Kawai et al., 2009). In addition, a morphology similar to that of *mreB* mutants has been observed in cells lacking the regulatory protein GlmR that enhance the glutamine-fructose-6-phosphate transaminase activity of GlmS to facilitate the diversion of carbon from fructose-6-phosphate to peptidoglycan synthesis (Foulquier et al., 2011). Removal of the mannose phosphate isomerase enzyme ManA is also accompanied with cell shape and cell wall integrity defects (Elbaz and Ben-Yehuda, 2010). Collectively, these observations illustrate the complexity of the cell wall homeostasis network, in which many components – some still unknown – are at play.

Targeting proteostasis has been proposed as a novel antibacterial strategy (Khodaparast et al., 2021). Based on the reduced tolerance of the *dnaK tig* double mutant to cell wall antibiotics, inducing proteostasis collapse while administering cell wall antibiotics may be an attractive combined strategy to increase treatment efficacy in the fight of pathogens. Finally, the *dnaK tig B. subtilis* mutants offer the potential to investigate direct or indirect links between cell wall integrity and cell shape, uncovering new components of the cell wall homeostasis network, and to study specific roles of DnaK and TF in *B. subtilis*, similar to the *E. coli* double mutant.

## Data availability statement

The original contributions presented in the study are included in the article/Supplementary material, further inquiries can be directed to the corresponding author.

## Author contributions

JM: conceptualization, data curation, formal analysis, investigation, methodology, validation, visualization, and writing-original draft. JH: conceptualization, data curation, formal analysis, investigation, methodology, and visualization. CW: project administration, funding acquisition, conceptualization, supervision, resources, investigation, and writing-editing and review. All authors contributed to the article and approved the submitted version.

## Funding

This work was funded by grant 2019-05578_3 from the Swedish Research Council.

## Conflict of interest

The authors declare that the research was conducted in the absence of any commercial or financial relationships that could be construed as a potential conflict of interest.

## Publisher’s note

All claims expressed in this article are solely those of the authors and do not necessarily represent those of their affiliated organizations, or those of the publisher, the editors and the reviewers. Any product that may be evaluated in this article, or claim that may be made by its manufacturer, is not guaranteed or endorsed by the publisher.
