# Supplementary material for: Bacillus subtilis forms twisted cells with cell wall integrity defects upon removal of the molecular chaperones DnaK and trigger factor
Source: Front Microbiol. 2023 Jan 16;13:988768. doi: 10.3389/fmicb.2022.988768 (PMC9886141; doi:10.3389/fmicb.2022.988768)
Supplement: Supplementary file 1 [file Table_1.DOCX]

**Supplementary Table 1.** Oligonucleotides used in this study

| **Oligonucleotide** | **Sequence (5’ 🡪 3’)** |
| --- | --- |
| AMYBACK1 | GACAATATCAGCATCCTTGCAGGGTATG |
| AMYFRONT1 | CAAAATTCTCCAGTCTTCACATCGGTTTG |
| AMYBACK3 | CTTTCGGTAAGTCCCGTCTAGCC |
| GFPMUT2_1 | CTCCAGTGAAAAGTTCTTCTCCTTTACTCATAATGTGACTTTCCTCCTTAAGCTTG |
| GFPMUT2_2 | ATGAGTAAAGGAGAAGAACTTTTCACTGGAG |
| DnaKNeongreen1 | CGAACTGTACAAAGGTGGTGGTGGTGGTGGTGGTGCTAGTAAAGTTATCGGAATCGACTTAGG |
| DnaKNeongreen2 | CTTGCATGCGGCTAGCTGTCTTATTTTTTGTTTTGGTCGTCG |
| DNAK2 | GGTTAAGCTTAAGGAGGTGATCTAGATGAGTAAAGTTATCGGAATCGACTTAGG |
| TIG1b | GGGTTAATTAAATCGCGCCATATAGTTGAAAGCG |
| TIG4 | CCCGGATCCCAGCCCTGCATTAACTATATTGCCC |
| TIG_FRAG1REV | TTTTCCCATTTTACAGACATCTAGATCACCTCCTTAAGCTTAATTGTTATCCG |
| TIG_FOR_2 | CGTTAAGCTAATTCGGTGGAAACGAGGTC |
| TIG_REV_2 | CTCGTTTCCACCGAATTAGCTTAACGGTTTTCTACAAGAAAATCAATTGCTTTG |
| TIG_FRAG2FOR | AGCTTAAGGAGGTGATCTAGATGTCTGTAAAATGGGAAAAACAAGAAGGC |
| ZAPA1 | GAACTGTACAAAGGTGGTGGTGGTGGTGGTGGTTCTGACGGCAAAAAAACAAAAACAAC |
| ZAPA2 | CCCGTCGACTCAATCCTTTTCTTTAAGCTGACGC |
