# Supplementary material for: Bacillus subtilis forms twisted cells with cell wall integrity defects upon removal of the molecular chaperones DnaK and trigger factor
Source: Front Microbiol. 2023 Jan 16;13:988768. doi: 10.3389/fmicb.2022.988768 (PMC9886141; doi:10.3389/fmicb.2022.988768)
Supplement: Supplementary file 7 [file Presentation_1.PDF]

Supplementary Material

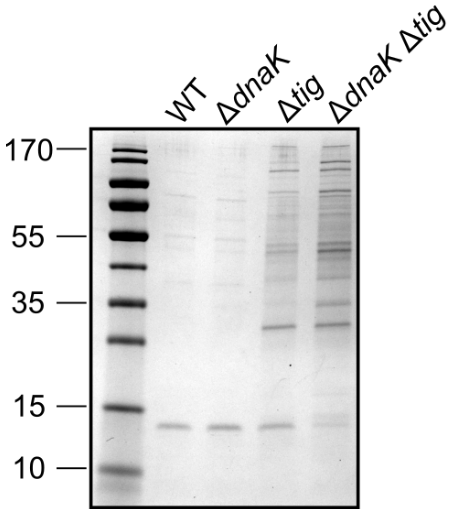

Supplementary Figure 1. Protein aggregates in strains grown at 37°C. Proteins were separated by SDS-PAGE and the gel stained with Coomassie blue. The Thermo Scientific™ PageRuler™ Prestained Protein Ladder was used as a size marker (with 10, 15, 25, 35, 40, 55, 70, 100, 130, and 170 kDa bands as molecular standards; only some are indicated on the left).

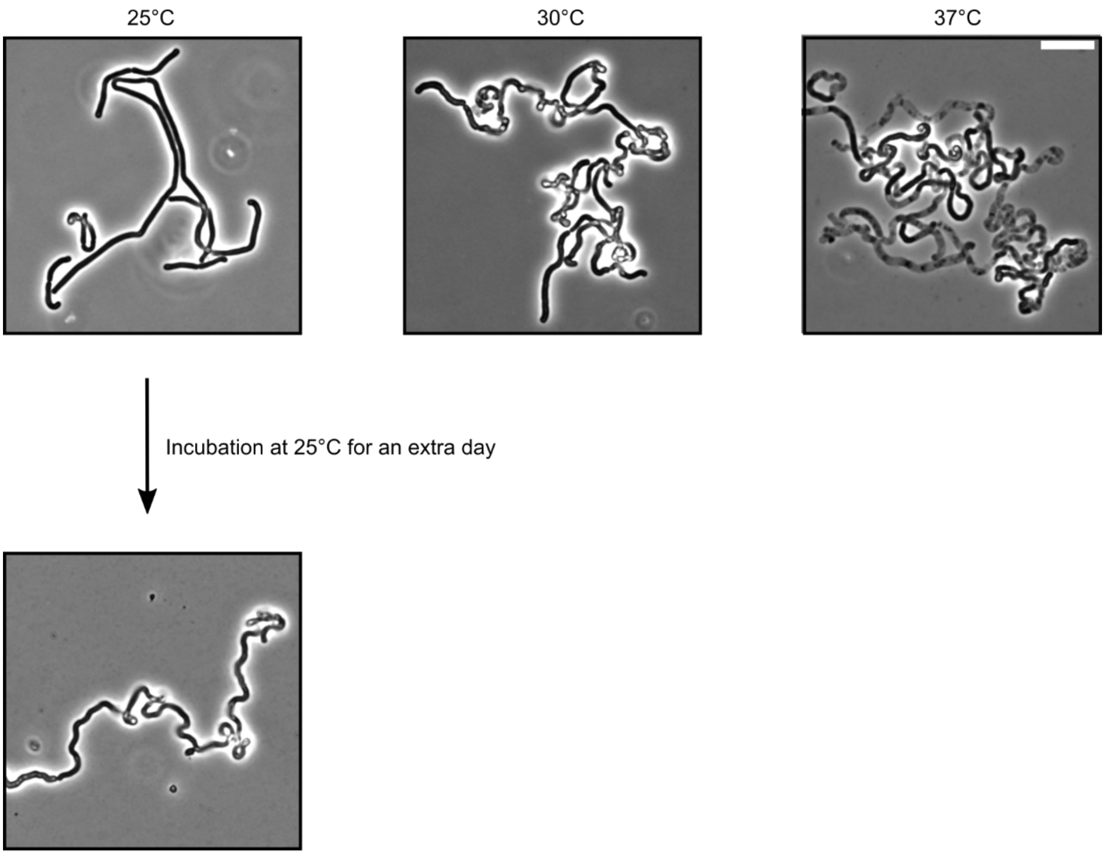

Supplementary Figure 2. Morphology of the *dnaK tig* double mutant at different temperatures. Representative phase contrast micrographs of Δ*dnaK* Δ*tig* (LUW878) cells taken from overnight TBAB plates at 25, 30, and 37°C. At 25°C, the morphology was not twisted after an overnight incubation, but cells became twisted when incubated for an extra day. The scale bar represents 10 µm.

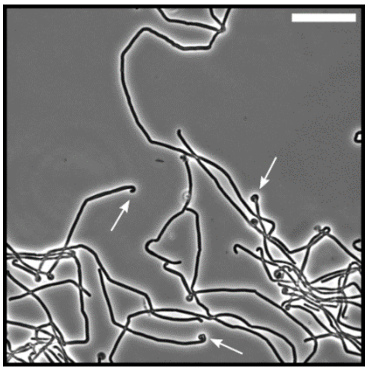

Supplementary Figure 3. Morphology of LUW878 from liquid cultures. Micrograph of *B. subtilis* Δ*dnaK* Δ*tig* (LUW878) from mid-exponential growth phase grown in NSMPG. Twisted filament ends are indicated by arrows. The scale bar represents 20 µm.

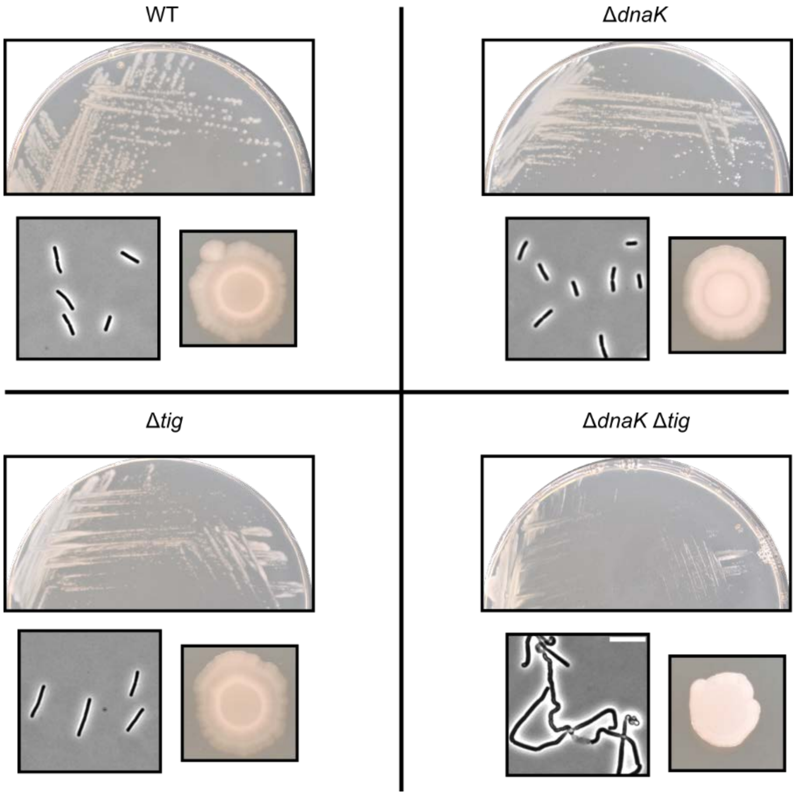

Supplementary Figure 4. Colony size, cell morphology, and spreading on NSMPG plates. Depiction of single colonies of *B. subtilis* wild-type (1A1), Δ*dnaK* (LUW876), Δ*tig* (LUW901), and Δ*dnaK* Δ*tig* (LUW878) from overnight NSMPG plates incubated at 30°C. Strains were streaked for single colonies from the glycerol stocks at -80°C. Below each plate image are representative phase contrast micrographs of the indicated strains. Cells were taken from single colonies from the overnight NSMPG plates. The scale bar represents 10 µm. Also shown are NSMPG plates inoculated with 5 µl drops of cells (from mid-exponential growth phase LB cultures) and incubated overnight at 30°C.

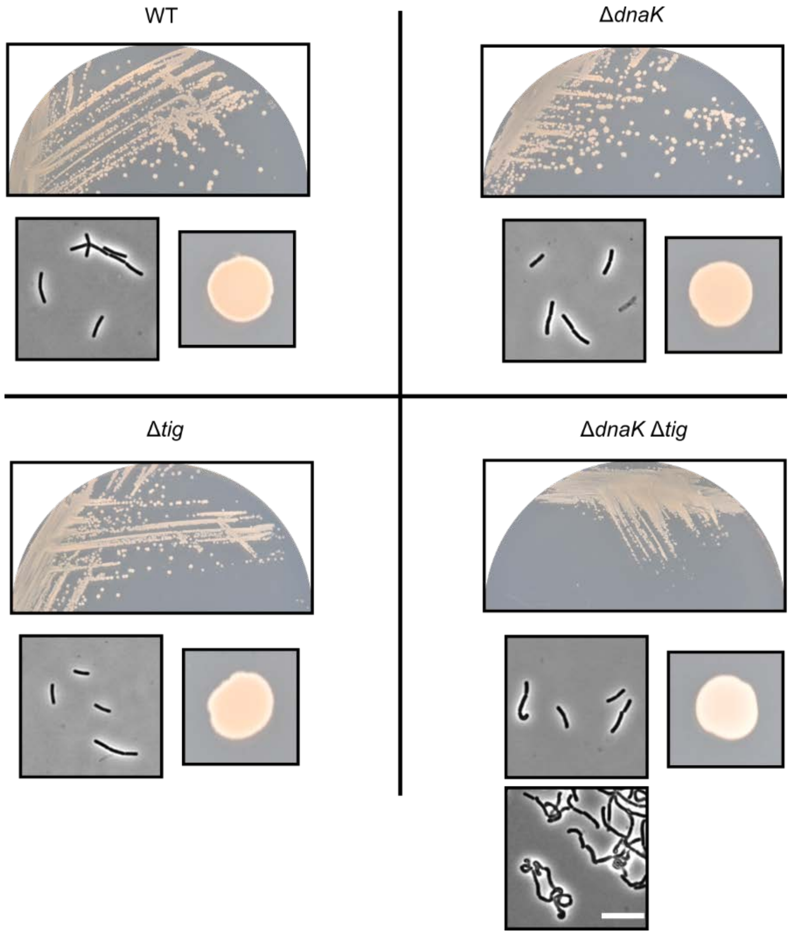

Supplementary Figure 5. Colony size, cell morphology, and spreading on minimal medium supplemented with glucose. Depiction of single colonies of *B. subtilis* wild-type (1A1), Δ*dnaK* (LUW876), Δ*tig* (LUW901), and Δ*dnaK* Δ*tig* (LUW878) from overnight plates (minimal medium supplemented with 0.5% glucose). Strains were streaked for single colonies from the glycerol stocks at -80°C. Below each plate image are representative phase contrast micrographs of the indicated strains. Cells for microscopy were taken from single colonies from the overnight plates. The scale bar represents 10 µm. Also shown are minimal medium plates (supplemented with glucose) inoculated with 5 µl drops of cells (from mid-exponential growth phase LB cultures) and incubated overnight at 30°C.

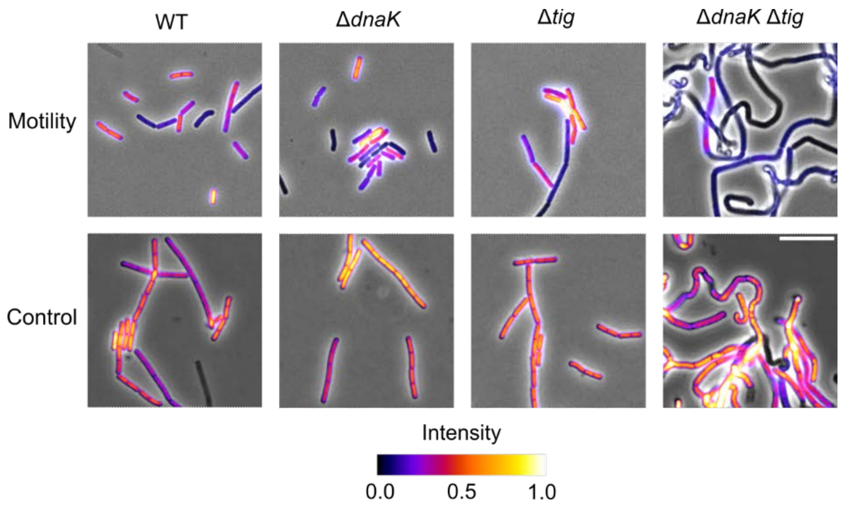

Supplementary Figure 6. Motility expression in single cells. Representative micrographs of *B. subtilis* wild-type (1A1), Δ*dnaK* (LUW876), Δ*tig* (LUW901), and Δ*dnaK* Δ*tig* (LUW878) strains expressing P_{hag}-*gfp* (top panels) as a reporter for motility and P_{const}-*gfp* (bottom panels) as a control. Cells were grown until mid-exponential growth phase in NSMPG. Shown are overlays of the phase contrast and fluorescence channel. Scale bar represents 10 µm.

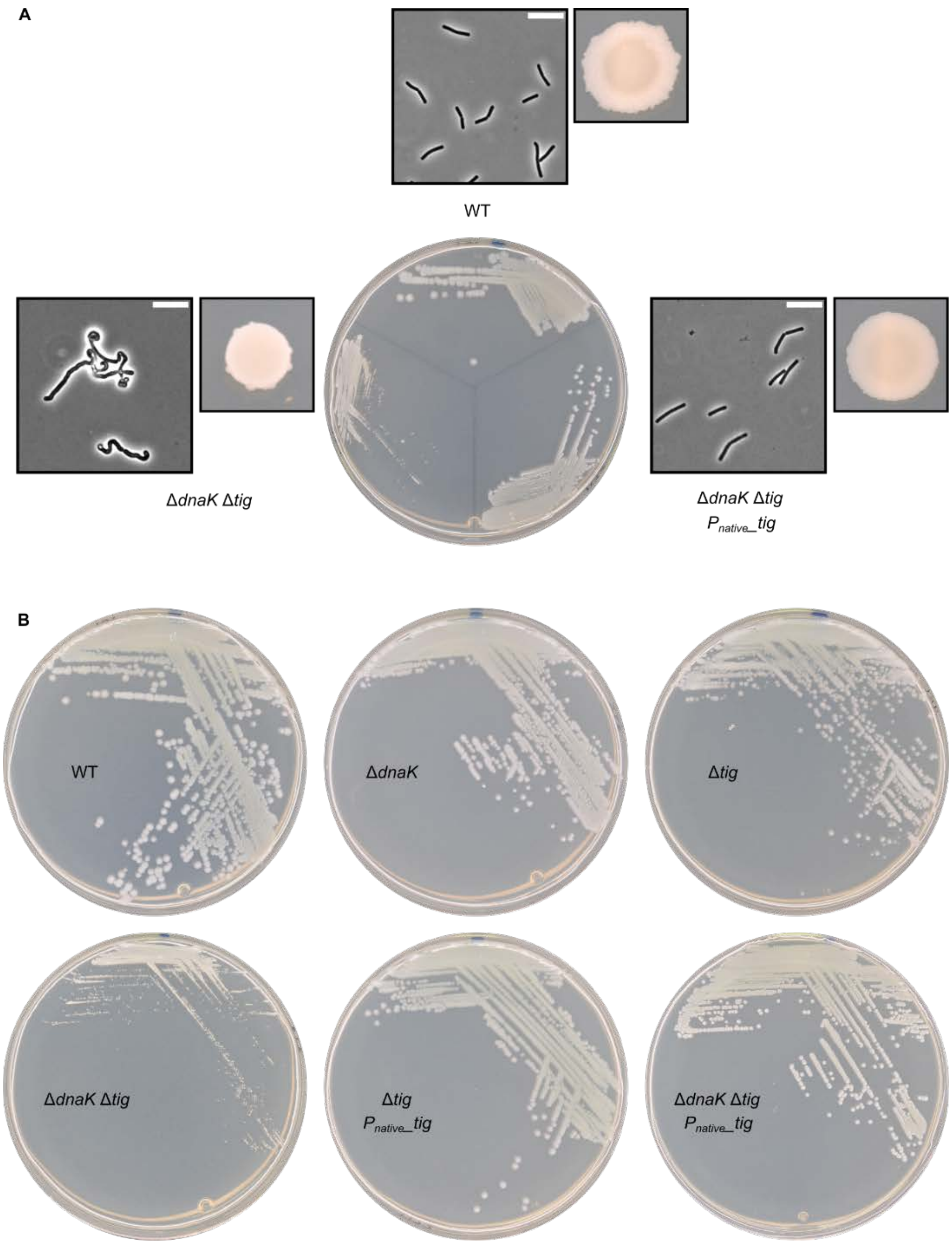

Supplementary Figure 7. Colony size, cell morphology, and spreading of *B. subtilis* Δ*dnaK* Δ*tig* after complementation with the *tig* gene under its native promoter. (**A**) TBAB plate containing *B. subtilis* wild-type (1A1), Δ*dnaK* Δ*tig* (LUW878), and Δ*dnaK* Δ*tig* complemented with the *tig* gene placed under its native promoter in the *amyE* locus (LUW1175). Strains were streaked for single colonies from the glycerol stocks at -80°C. Also shown are representative phase contrast micrographs of the indicated strains. Cells for microscopy were taken from single colonies from overnight plates. The scale bar represents 10 µm. To the right of phase contrast images are TBAB plates inoculated with 5 µl drops of cells (from mid-exponential growth phase LB cultures) and incubated overnight at 30°C. (**B**) TBAB plates containing *B. subtilis* wild-type (1A1), Δ*dnaK* (LUW876), Δ*tig* (LUW901), Δ*dnaK* Δ*tig* (LUW878), Δ*tig* complemented with the *tig* gene placed under its native promoter (LUW1173), and Δ*dnaK* Δ*tig* complemented with the *tig* gene placed under its native promoter (LUW1175). Strains were streaked for single colonies from the glycerol stocks at -80°C onto TBAB plates.

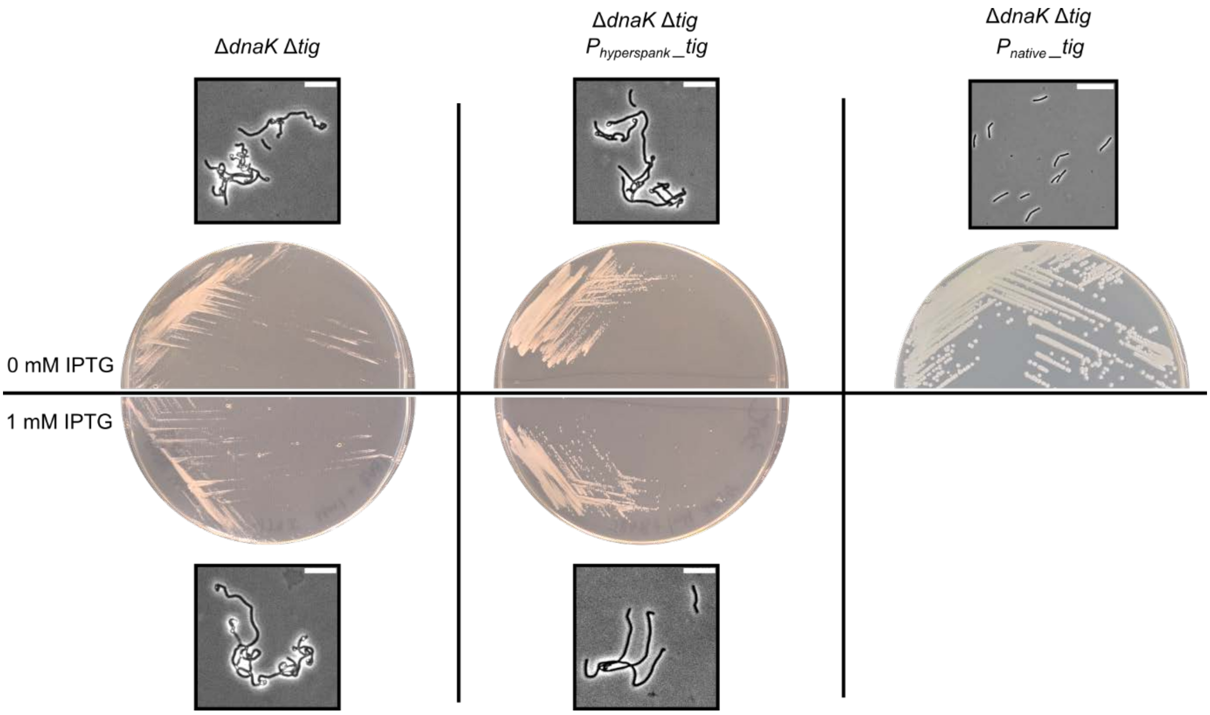

Supplementary Figure 8. Colony size and cell morphology of *B. subtilis* Δ*dnaK* Δ*tig* after complementation with the *tig* gene under the $P_{hyperspank}$ promoter. TBAB plates without (upper row) or with 1mM IPTG (lower row) containing *B. subtilis* Δ*dnaK* Δ*tig* (LUW878), Δ*dnaK* Δ*tig* complemented with the *tig* gene placed under the IPTG-inducible $P_{hyperspank}$ promoter (LUW1150), and Δ*dnaK* Δ*tig* complemented with the *tig* gene placed under its native promoter in the *amyE* locus (LUW1175). Strains were streaked for single colonies from the -80°C freezer. Also shown are representative phase contrast micrographs of the indicated strains. Cells were taken from single colonies from overnight plates. The scale bar represents 20 µm.

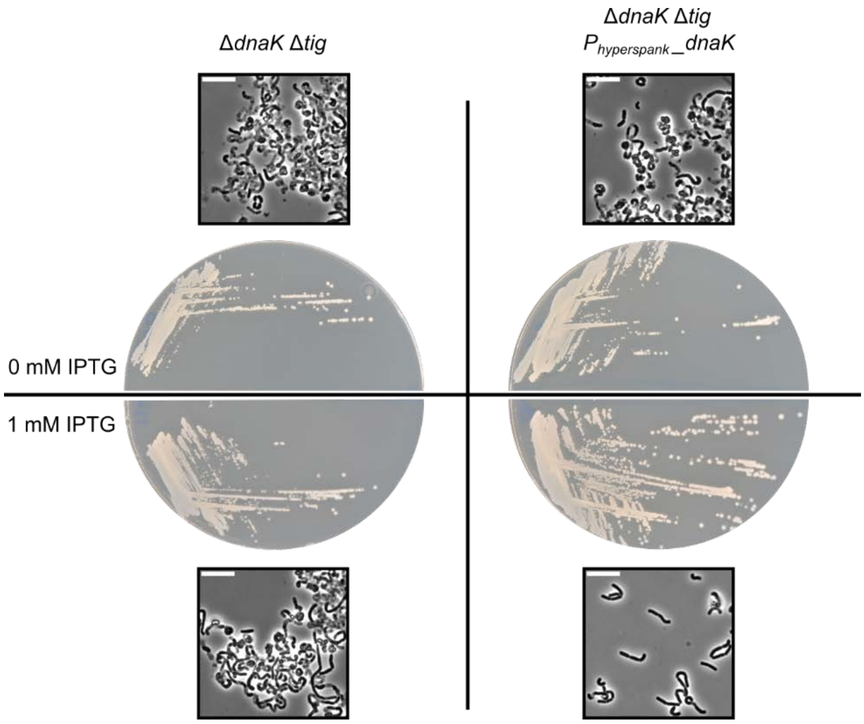

Supplementary Figure 9. Colony size and cell morphology of *B. subtilis* Δ*dnaK* Δ*tig* after complementation with the *dnaK* gene under the $P_{hyperspank}$ promoter. TBAB plates without (upper row) or with 1mM IPTG (lower row) containing *B. subtilis* Δ*dnaK* Δ*tig* (LUW878), and Δ*dnaK* Δ*tig* complemented with the *tig* gene placed under the IPTG-inducible $P_{hyperspank}$ promoter (LUW907). Strains were streaked for single colonies from the glycerol stocks at -80°C and incubated at 30°C for two days. Also shown are representative phase contrast micrographs of the indicated strains. Cells for microscopy were taken from single colonies from the incubated plates. The scale bar represents 10 µm.

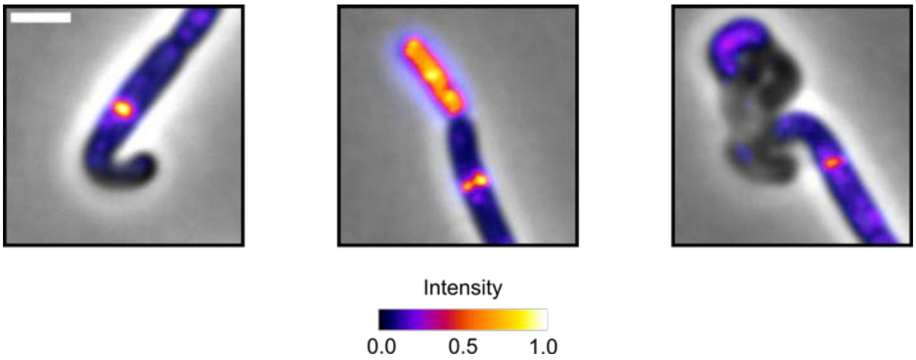

Supplementary Figure 10. De-localized mNG-ZapA in the aberrant filament ends of *B. subtilis* Δ*dnaK* Δ*tig*. Shown are overlays of the phase contrast and fluorescence micrographs. Cells were grown until mid-exponential growth phase in NSMPG. Scale bar represents 2 µm.

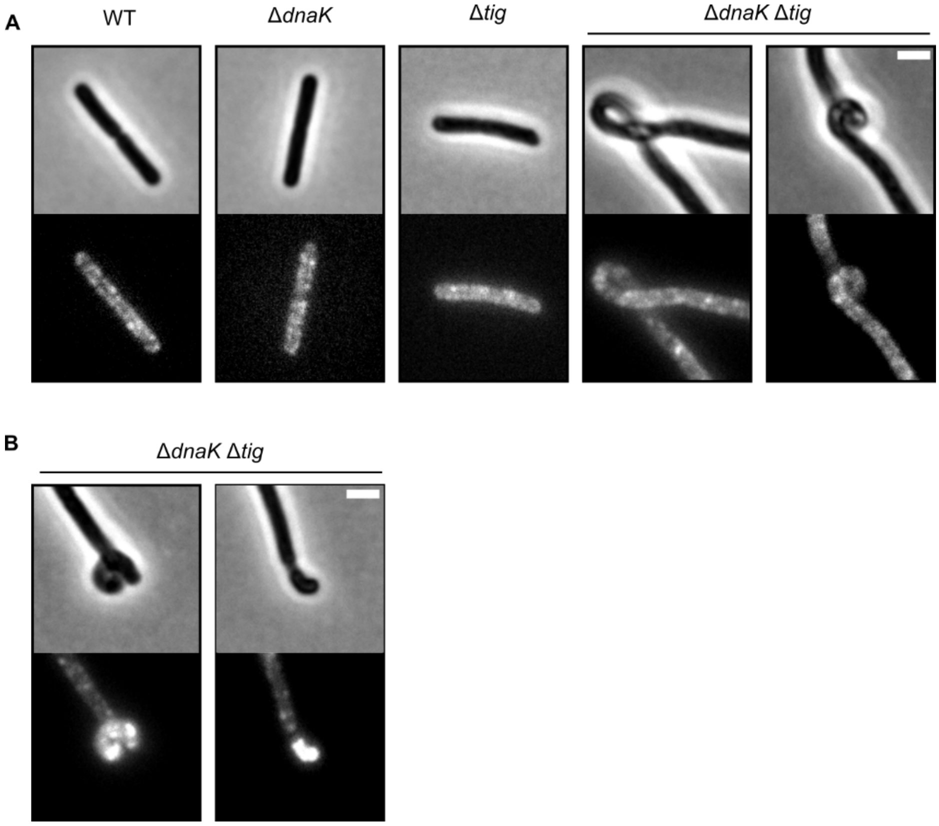

Supplementary Figure 11. MreB localization pattern. (**A**) Phase contrast and fluorescence micrographs of the indicated strains expressing mCherry-MreB. Cells were grown until mid-exponential growth phase in NSMPG. Scale bar represents 2 µm. (**B**) Phase contrast and fluorescence micrographs of the *dnaK tig* double mutant expressing mCherry-MreB, with focus on the aberrant filament ends. Scale bar represents 2 µm.

frontiers

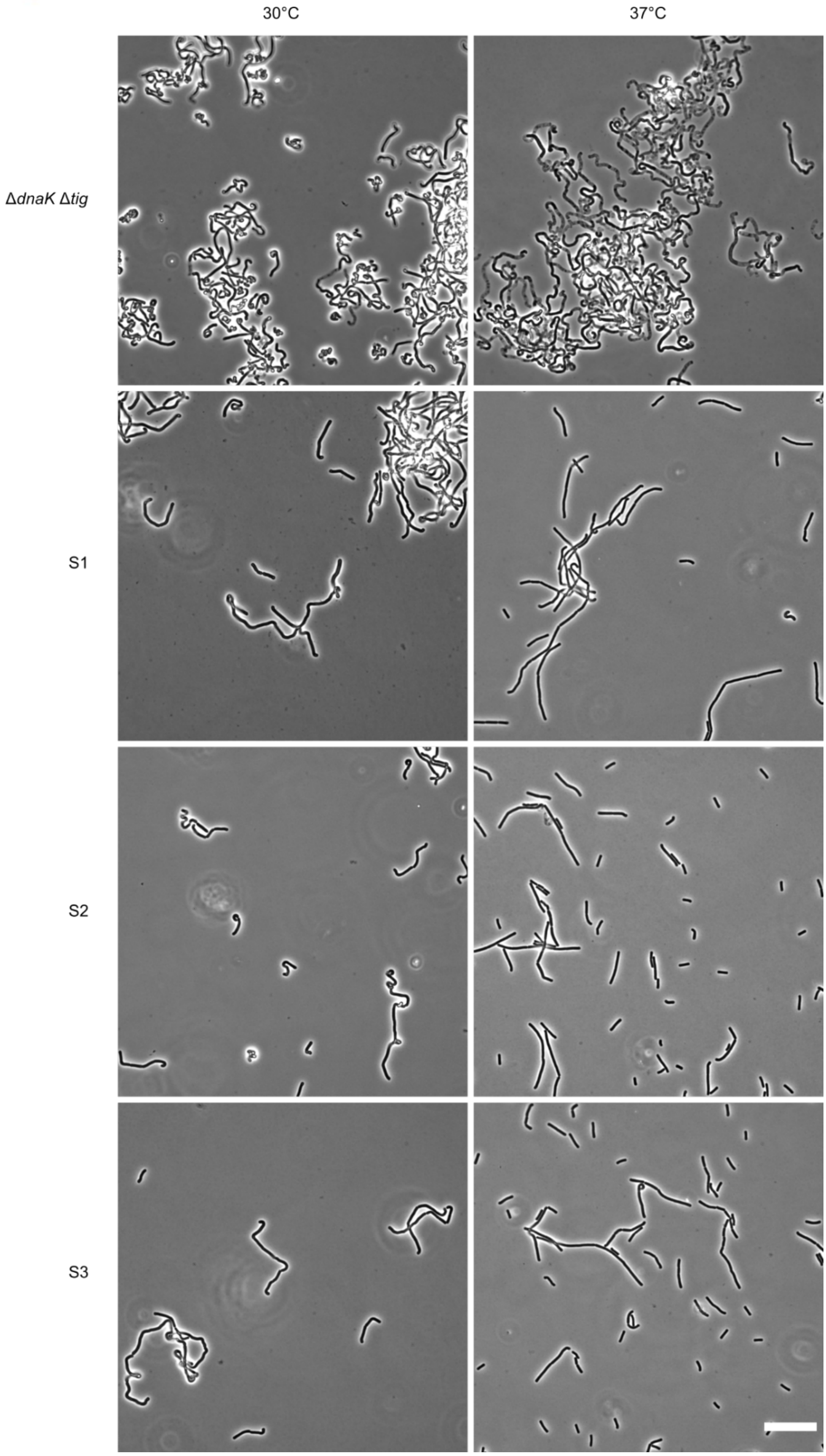

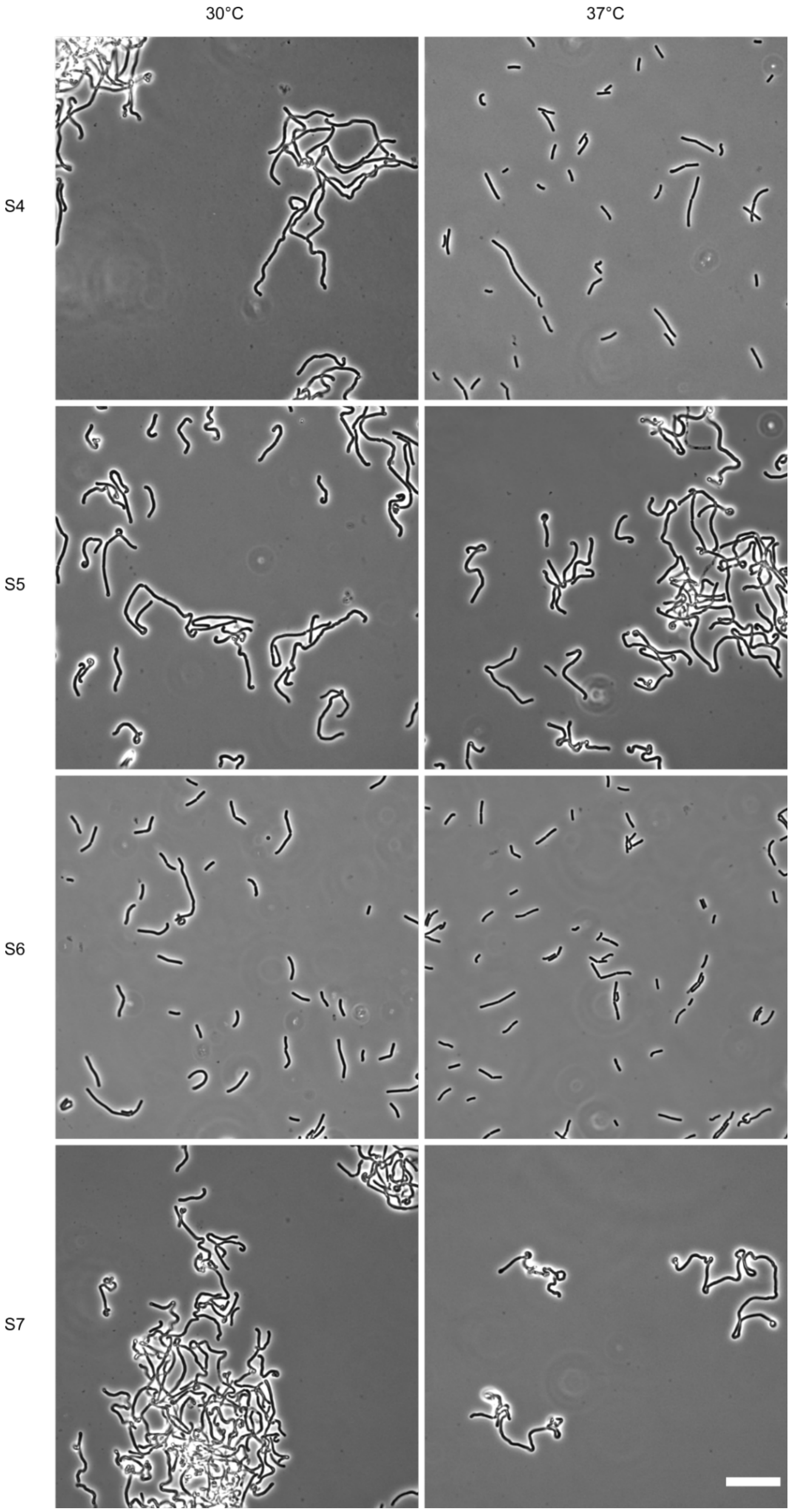
30°C
37°C
S4
S5
S6
S7

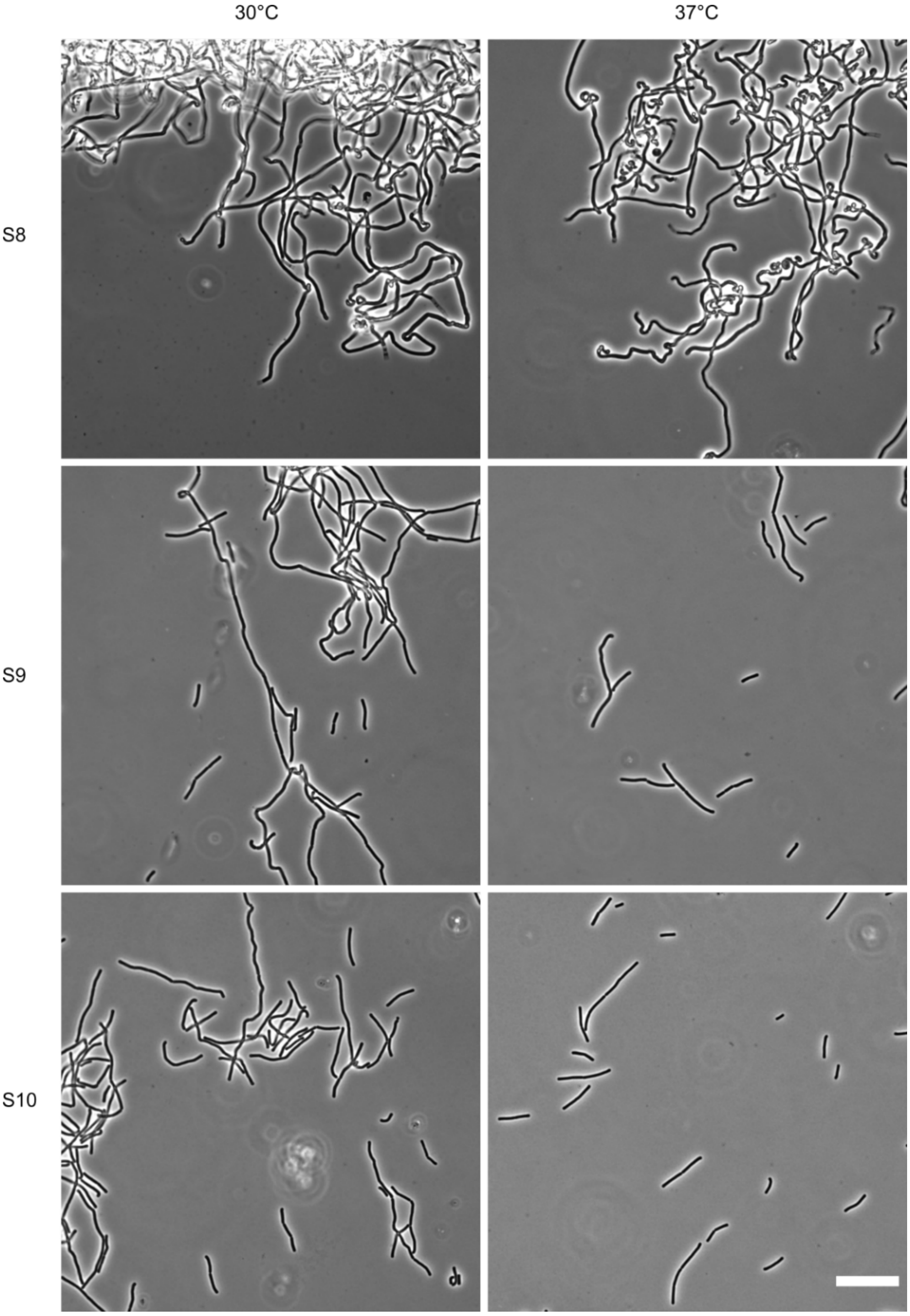

Supplementary Figure 12. Representative phase contrast whole frame micrographs of LUW878 (Δ*dnaK* Δ*tig*) and the ten isolated LUW878 suppressors. Cells were grown overnight on TBAB plates incubated at 30 or 37°C. Scale bar represents 20 µm.
